# The role of hydrophobic interactions in positioning of peripheral proteins in membranes

**DOI:** 10.1186/1472-6807-7-44

**Published:** 2007-06-29

**Authors:** Andrei L Lomize, Irina D Pogozheva, Mikhail A Lomize, Henry I Mosberg

**Affiliations:** 1 Department of Medicinal Chemistry, College of Pharmacy, University of Michigan, Ann Arbor, MI 48109-1065, USA; 2 College of Literature, Science and the Arts, University of Michigan, Ann Arbor, MI 48109-1065, USA

## Abstract

**Background:**

Three-dimensional (3D) structures of numerous peripheral membrane proteins have been determined. Biological activity, stability, and conformations of these proteins depend on their spatial positions with respect to the lipid bilayer. However, these positions are usually undetermined.

**Results:**

We report the first large-scale computational study of monotopic/peripheral proteins with known 3D structures. The optimal translational and rotational positions of 476 proteins are determined by minimizing energy of protein transfer from water to the lipid bilayer, which is approximated by a hydrocarbon slab with a decadiene-like polarity and interfacial regions characterized by water-permeation profiles. Predicted membrane-binding sites, protein tilt angles and membrane penetration depths are consistent with spin-labeling, chemical modification, fluorescence, NMR, mutagenesis, and other experimental studies of 53 peripheral proteins and peptides. Experimental membrane binding affinities of peripheral proteins were reproduced in cases that did not involve a helix-coil transition, specific binding of lipids, or a predominantly electrostatic association. Coordinates of all examined peripheral proteins and peptides with the calculated hydrophobic membrane boundaries, subcellular localization, topology, structural classification, and experimental references are available through the Orientations of Proteins in Membranes (OPM) database.

**Conclusion:**

Positions of diverse peripheral proteins and peptides in the lipid bilayer can be accurately predicted using their 3D structures that represent a proper membrane-bound conformation and oligomeric state, and have membrane binding elements present. The success of the implicit solvation model suggests that hydrophobic interactions are usually sufficient to determine the spatial position of a protein in the membrane, even when electrostatic interactions or specific binding of lipids are substantial. Our results demonstrate that most peripheral proteins not only interact with the membrane surface, but penetrate through the interfacial region and reach the hydrocarbon interior, which is consistent with published experimental studies.

## Background

More than half of all proteins interact with membranes. These proteins can be classified as transmembrane, integral monotopic, or peripheral. Transmembrane proteins comprise numerous receptors, channels, transporters, photosystems, and respiratory complexes. Integral monotopic proteins associate with the membrane permanently, but do not traverse the lipid bilayer. Peripheral proteins are water-soluble and associate with lipid bilayers reversibly. They include numerous membrane-associated enzymes, transporters, signaling lipid-binding domains (C1, C2, PH, FYVE, PX, ENTH, ANTH, FERM, etc.), antibacterial peptides, hormones, toxins, pulmonary surfactant-associated polypeptides, peptaibols, lipopeptides, etc. [2-4]. Experimental three-dimensional (3D) structures are currently available for hundreds of membrane-associated proteins; however, their precise spatial positions in the lipid bilayer are usually unknown. The arrangement of proteins in membranes may affect their conformation, biological activity, folding, thermodynamic stability, and binding of surrounding macromolecules and substrates [2,5].

Spatial positions in the lipid bilayer have been experimentally studied for approximately 50 peripheral proteins with known three-dimensional (3D) structures using site-directed spin labeling, chemical labeling, measurement of membrane binding affinities of protein mutants, fluorescence spectroscopy, solution or solid-state NMR spectroscopy, ATR FTIR spectroscopy, or X-ray diffraction. In many cases, some of the membrane-embedded residues have been identified (Tables 1). Membrane-docking geometries of four C2 domains, monomeric EEA1 FYVE domain, and secreted phospholipase A2 have been defined from spin-labeling or other experimental data [6-10], although the coordinates of the proteins with lipid bilayer boundaries are publicly available only for the human pancreatic phospholipase A2 [11].

**Table 1:** Comparison of membrane-bound residues in peripheral proteins according to experimental studies and calculations peripheral proteins.

		**Experiment^a^**			**Calculation^b^**		
		
**Protein name**	**PDB id**	**Residues buried from water or important for membrane binding**	**Method**	**References**	**Δ*G*_*calc *_(kcal/mol)**	***D *(Å)**	**Residues penetrating the hydrocarbon membrane core**
**Lipid clamps (specific binding of lipid ligands)**							

C2 domain of phospholipase A2	1rlw	**A34, F35, G36, M38, L39, Y96, V97, M98**	Bn, SL	[6, 84, 85]	-7.1	5.3	**A34, F35, G36, M38, L39, Y96, V97, M98**
C2A domain of synaptotagmin I	1byn	**M173, G174, F234**	Bn, SL	[7, 86]	-4.4	3.7	**M173, G174, F234**
C2 domain of protein knase Cα	1dsy	**N189**, R249, R252	SL	[8]	-2.0	1.5	P188, **N189**, T250
C2B domain of synaptotagmin I	1uov	**G305, I367**	SL	[9]	-4.3	2.4	V304, **G305, I367**, G368
C2 domain of protein kinase Cε	1gmi	**I89**, Y91	Bn	[87]	-5.1	2.4	V29, P31, **I89**
PX domain (p40phox)	1h6h	**F35, Y94, V95**	Bn	[39]	-4.5	3.2	**F35, Y94, V95**
PX domain (p47phox)	1o7k	**W80**	Bn	[39]	-1.9	1.4	**W80**
FYVE domain of EEA1	1hyi	**V21, T22**	NMR	[40]	-2.9	2.5	**V21, T22**
FYVE domain of Vps27p	1vfy	**L185, L186**	Bn	[88]	-4.0	2.9	**L185, L186**
C1 domain of protein kinase Cδ	1ptr	**W252, L254, V255**	Bn	[89]	-5.7	6.8	M239, P241, L250, **W252**, G253, **L254, V255**
Epsin ENTH domain	1h0a	**L6, M10**	Bn	[77]	-5.2	2.9	**L6, M10**, I13, V14
Discoidin domain of factor V	1czs	**W26, W27**	Bn	[90]	-3.0	4.2	**W26, W27**, L79, and S80
Discoidin domain of factor Va	1sdd	**Y1956, L1957, W2063, W2064**	Bn	[91]	-5.6	3.3	**Y1956, L1957, W2063, W2064**
Discoidin domain of factor VIII	1d7p	**M2199, F2200, L2251, L2252**	Bn	[92]	-8.1	3.9	**M2199, F2200, L2251, L2252**
Annexin V	1a8a	**T72**, S144**, W185**, S228, S303	SL	[93]	-6.3	2.5	L29, **T72**, A101, **W185**, A260
Annexin XII	1dm5	E142, S144, G145	SL	[94]	-8.3	3.1	I29, L101, I185
Equinatoxin II	1iaz	**W112**	NMR	[95]	-2.2	3.1	P81, V82, **W112**

**Other proteins**							

Prostaglandin H2 synthase 1	1q4g	**I74, W75, W77, L78, F88, F91, L92, W98, L99, F102**	Bn, Fn	[96]	-37.8	7.2	**I74, W75, W77, L78**, T81, L82, **F88, F91, L92, W98, L99, F102**, V103, T106, F107, I108
Antimicrobial peptide kalata B1	1nb1	**W19-V21, L27-V29**	NMR	[97]	-5.4	5.2	**W19-V21**, **L27-V29**
Pancreatic phospholipase A_2_, group IB	4p2p	**W3**	Flq	[11]	-8.7	3.5	**W3**, H17, L19, M20, L64, V65
Bee venom phospholipase A_2_	1poc	**I2**, K14, **I78**	SL*	[42]	-10.3	5.7	I1, **I2**, Y3, P4, G5,**I78**, F82, M86, L90
Human secretory phospholipase A_2_, group IIa	1n28	**V3**, K10, **L19, F23, F63**	SL*	[43]	-6.6	4.8	**V3, L19, F23, F63**
Snake venom phospholipase A_2_, group I	1poa	**W61, F64**, Y110	Bn	[98]	-5.4	4.5	Y3, W18, W19, **W61, F64**
Snake venom phospholipase A_2_, group II	1vap	W20, **W30, W109**	FL	[99]	-10.2	4.3	F3, M13, L19, **W30**, M61, **W109**
Snake venom phospholipase A_2_, group IIB	1jia	Y120, **P121, I124**, L125	Bn	[100]^d^	-8.7	3.1	V20, F24, A119, **P121, I124**
Phospholipase C	2ptd	**I43, W47, W242**	Bn	[101]	-6.0	3.9	P42, **I43**, **W47**, T240, A241, **W242**
α-toxin (bacterial phospholipase C)	1ca1	**Y331, F334**	Fn	[102]	-4.5	2.2	V143, A146, M210, W214, **Y331, F334**
15-lipoxygenase	1lox	Y15, F70, **L71**, *W181*^c^, **L195**	Bn	[103]	-7.4	6.3	**L71**, I194, **L195**, L291, Y292, F412
8R-lipoxygenase	1zq4	**W413, W449**	FL, Fn	[104]	-5.0	5.9	**W413**, F414, Y448, **W449**, V560, M570
Cholesterol oxidase	1coy	**M81**	FLq	[105]	-4.1	5.3	**M81**, M332, W333, L369, Y372
Signal peptidase	1kn9	**W300**	Fn	[106]	-4.5	4.5	**W300**, M301, F303, L314, L316
Synapsin I	1auv	Regions 166–192, 233–258, 278–327	CL	[107]	-4.4	2.5	**L168, V172, L297**
α-synuclein	1xq8	**T59, V63, G67, V70, V74, V77, T81, A85, A89**	SL	[108]	-22.8	17.9	**T59, V63**, V66, **G67**, **V70, V74, V77**, A78, **T81, A85**, I88, **A89**
Perfringolysin	1pfo	**W466**	Bn	[109]	-5.5	3.4	L462, **W466**, L491, Y492
Daptomycin	1t5n	**Kyn14**	FL	[110]	-9.8	4.9	Dka1, W2, **Kyn14**
Lactoferricin B	1lfc	**W6, W8**	FL	[111]	-4.6	5.3	**W6, W8**, L13-P16, I18
Hanatoxin	1d1h	**W30**	FLq	[112]	-2.5	3.9	Y4, L5, F6, **W30**
Subtilosin	1pxq	**W34**	FL	[113]	-6.7	5.1	F22, F31, **W34**

^**a **^Bn – based on membrane binding affinities of mutants; SL – spin-labeling data based on depth parameters Φ (SL* – based only on exposure of labels to polar probes); FL – fluorescence; FLq – fluorescence quenching; Fn – functional studies of mutants; CL – chemical labeling. Underlined residues are located in the lipid headgroup region, close to the hydrophobic boundaries. Coinciding residues in two sets are shown as bold.

^**b **^Δ*G*_*calc*_, calculated binding energies (kcal/mol); *D *(Å) maximal penetration depths of atoms into the hydrocarbon core. Residues penetrating into lipid acyl chain region are identified based on locations of side-chain atoms or NH hydrogens (when compared with NMR data).

^**c **^W181 is missing in the crystal structure.

^**d **^Data for a homologous protein

Positions of proteins in membranes can also be determined computationally. Three major categories of computational methods can be used for this purpose: molecular dynamics simulations with explicit lipids [12,13], energy minimization of the protein in the hydrophobic slab using the implicit solvation model [14-16], or optimization of electrostatic interaction energy between cationic proteins and a negatively charged planar membrane surface [17-22]. Most computational studies have been conducted for α-helical peptides and transmembrane proteins. Spatial positions with respect to the membrane have been theoretically predicted and compared with experimental data only for a few proteins, such as toxins, membrane-targeting domains, viral matrix domains, phospholipases A2, and prostaglandine synthase ([13,16,19-22]. However, the coordinates of these proteins with their membrane boundaries are not available. Thus, it is difficult to compare the reliability and precision of different methods.

We report here the first large-scale computational analysis of peripheral proteins from the Protein Data Bank (PDB) [23]. Several hundreds of peripheral proteins and peptides were considered. Their spatial positions in the membrane were calculated, compared with available experimental data, and deposited in our OPM database [24]. The computational approach applied implements the commonly accepted model of the lipid bilayer in a fluid state, where the hydrocarbon core region formed by lipid acyl chains is surrounded by two interfacial regions formed by lipid head groups. The hydrocarbon region has well defined boundaries where the effective concentration of water changes from nearly zero to ~2 M at a distance of several angstroms [25]. The optimal rotational and translational position of each protein with respect to the lipid bilayer is determined by minimizing transfer energy of the protein from water to the membrane hydrocarbon core approximated by a nonpolar solvent decadiene. In this approximation, the binding of a protein to the membrane is driven by hydrophobic interactions and opposed by desolvation of polar and charged groups. This approach has been successfully tested for integral transmembrane proteins [26]. It has no training or adjustable parameters that must be optimized specifically for the proteins studied in the present work. All required atomic solvation parameters were previously determined from experimental water-decadiene transfer energies of model organic compounds [27].

Comparison with experimental studies demonstrated that the implicit solvent model performs well for 53 experimentally studied peripheral proteins and peptides with diverse 3D structures. Hence, this model was applied to several hundreds of other proteins whose spatial positions in membranes have not yet been experimentally evaluated. This analysis allows estimation of the contributions of hydrophobic interactions to membrane binding energies and identification of membrane-anchoring motifs of individual proteins, such as exposed non-polar loops, amphiphilic α-helices, β-hairpins, or non-covalently bound lipids.

## Results

### 1. Comparison with experimental studies of protein positions in membranes

**Figure 1:**
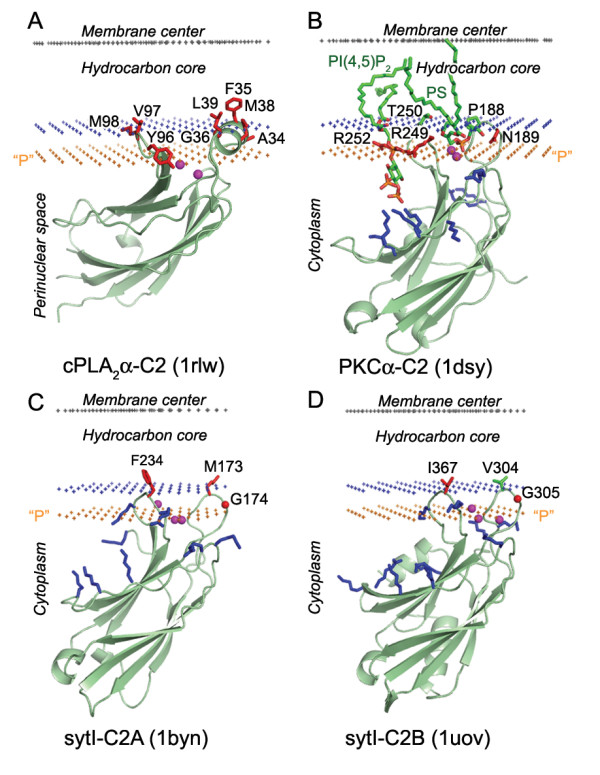
**Calculated membrane binding modes for C2 domains of cPLA_2α _(A), PKCα-C2 with lipid ligands PS and PI(4,5)P_2 _(B), sytI-C2A (C) and sytI-C2B (D)**. The backbone of C2 domains and the specific lipid ligands are shown in ribbon and stick models, respectively. Ca^2+ ^ions are shown as balls colored magenta. Residues identified as penetrating to a non-polar environment by SDSL are colored red. Cationic residues involved in ligand binding and membrane interactions are colored blue. The hydrocarbon core boundary at the cytoplasmic side is indicated by blue dots. The layer of lipid phosphates ("P") is shown by gold dots (at 5 Å outside the hydrocarbon boundary [28]). The center of the membrane is indicated by grey dots (at 15 Å inside the boundary). All Figures were generated by PyMol.

**Figure 2:**
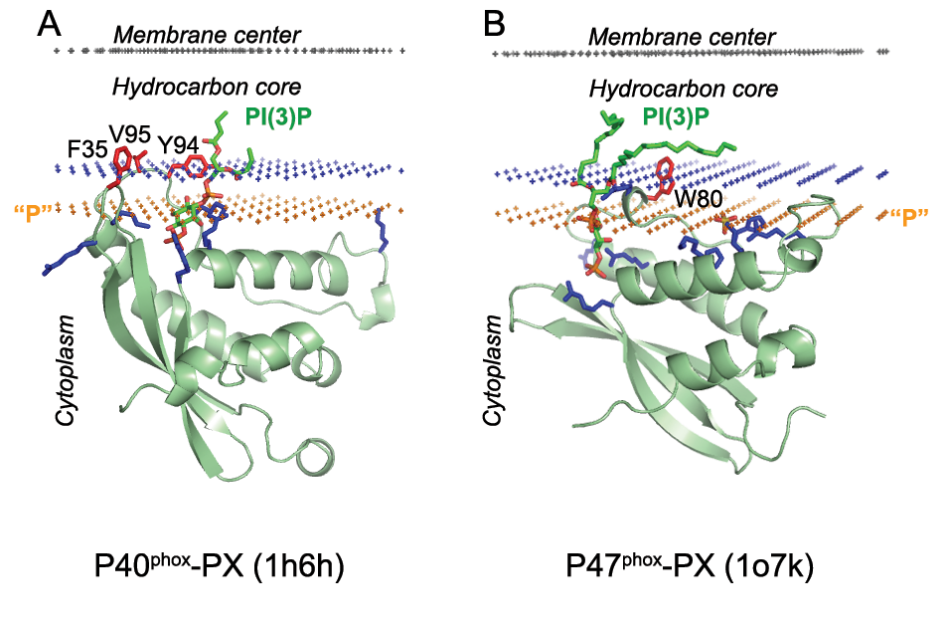
**Calculated membrane binding modes for PX domains of P40^**phox **^with lipid ligand PI(3)P (A) and P47^**phox **^with lipid ligand PI(3,4)P_2 _(B)**. The backbone of PX domains and the specific lipid ligands are shown in ribbon and stick model, respectively. Residues identified as penetrating to the membrane in mutagenesis and binding experiments are colored red. Cationic residues involved in ligand binding and membrane interactions are colored blue. Hydrocarbon core boundary at the cytoplasmic side is indicated by blue dots. The layer of lipid phosphates ("P") is shown by gold dots (at 5 Å outside the hydrocarbon boundary). The center of membrane is indicated by grey dots (at 15 Å inside the boundary).

**Figure 3:**
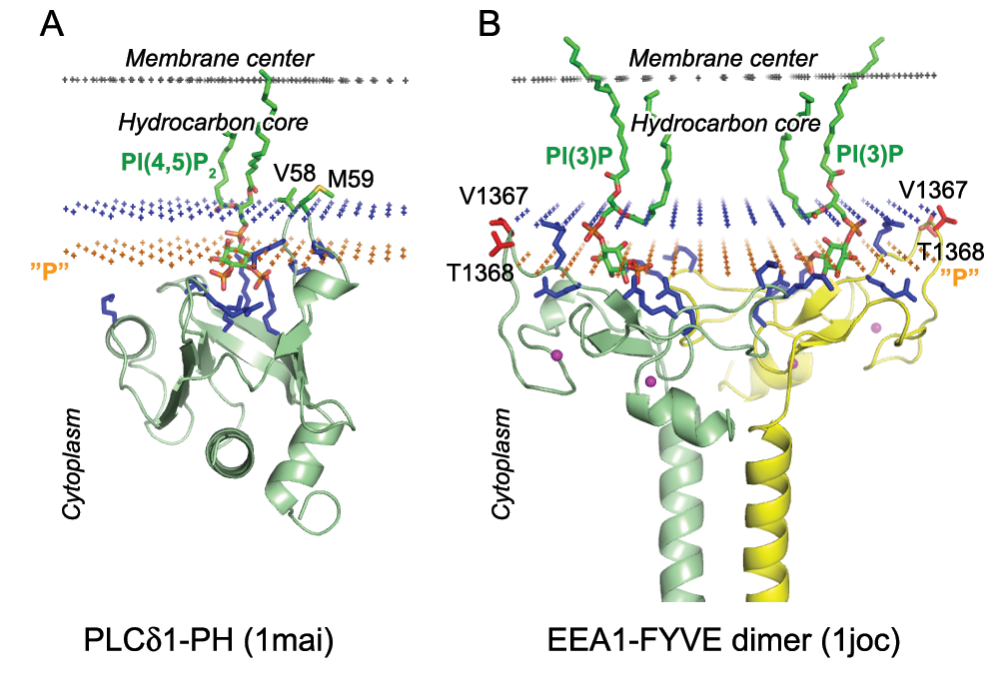
**Calculated membrane binding modes for PH domain of PLCδ1 with lipid ligand PI(4,5)P_2 _(A) and EEA1-FYVE domain with lipid ligand PI(3)P (B)**. The backbone of two domains and the specific lipid ligands are shown in ribbon and stick model, respectively. Residues identified as penetrating the non-polar interior of micelles by NMR (and also shown to be important for membrane binding) are colored red. Zn^2+ ^ions (FYVE domain) are shown as balls colored magenta. Cationic residues involved in ligand and membrane interactions are colored blue. Hydrocarbon core boundary at the cytoplasmic side is indicated by blue dots. The layer of lipid phosphates ("P") is shown by gold dots (at 5 Å outside the hydrocarbon boundary). The center of membrane is indicated by grey dots (at 15 Å inside the boundary).

**Figure 4:**
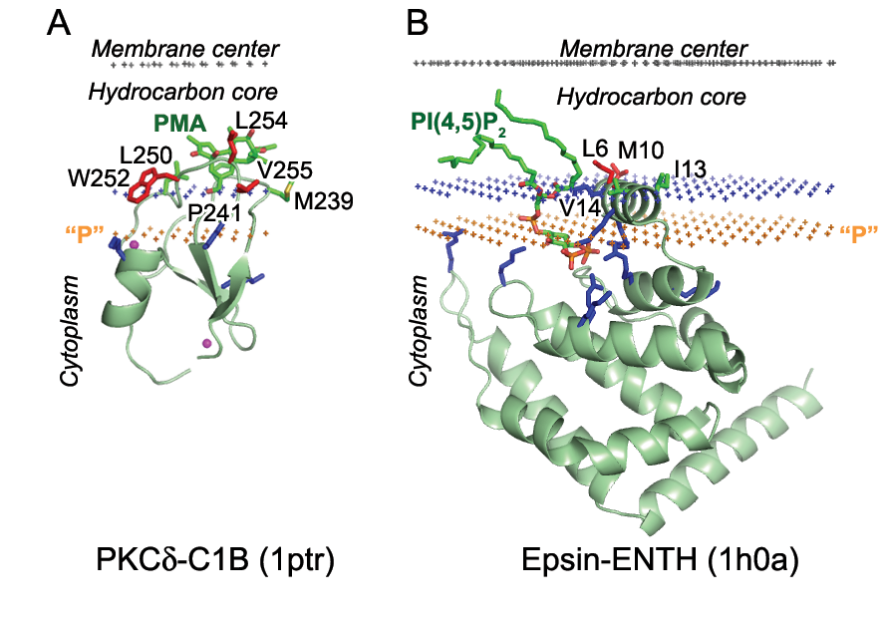
**Calculated membrane binding modes for C1B domain of PLCδ with phorbol ester PMA (A) and Epsin-ENTH domain with lipid ligand PI(4,5)P_2 _(B)**. The backbone of two H-type domains and the specific lipid ligands are shown in ribbon and stick model, respectively. Residues identified as penetrating to the membrane in mutagenesis and binding experiments are colored red. Zn^2+ ^ions (C1 domain) shown as balls colored magenta. Cationic residues involved in ligand and membrane interactions are colored blue. Hydrocarbon core boundary at the cytoplasmic side is indicated by blue dots. The layer of lipid phosphates ("P") is shown by gold dots (at 5 Å outside the hydrocarbon boundary). The center of membrane is indicated by grey dots (at 15 Å inside the boundary).

**Figure 5:**
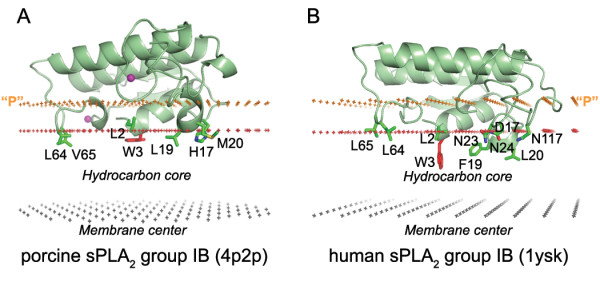
**Positions of two homologous phospholipases in the lipid bilayer**. (A) position of porcine pancreatic sPLA_2 _calculated by PPM, and (B) experimentally defined arrangement of human pancreatic sPLA_2_. (a homology model, 1ysk PDB entry) studied by ATR FTIR spectroscopy [11]. Trp residues identified as penetrating the non-polar environment by fluorescence quenching are colored red. Ca^2+ ^ions are shown as balls colored magenta. Hydrocarbon core boundary at the extracellular side is indicated by red dots. The layer of lipid phosphates ("P") is shown by gold dots (at 5 Å outside the hydrocarbon boundary). The center of membrane is indicated by grey dots (at 15 Å inside the boundary). The obtained orientations are quite similar, but the model of human sPLA_2 _(B) penetrates slightly deeper and with a slightly different (by ~10°) tilt into the membrane interior. Therefore, N23, N24, N117, which were localized outside hydrophobic boundaries by our method (A), appeared to be immersed into the hydrophobic slab in the experimentally-derived position of the protein (B).

**Figure 6:**
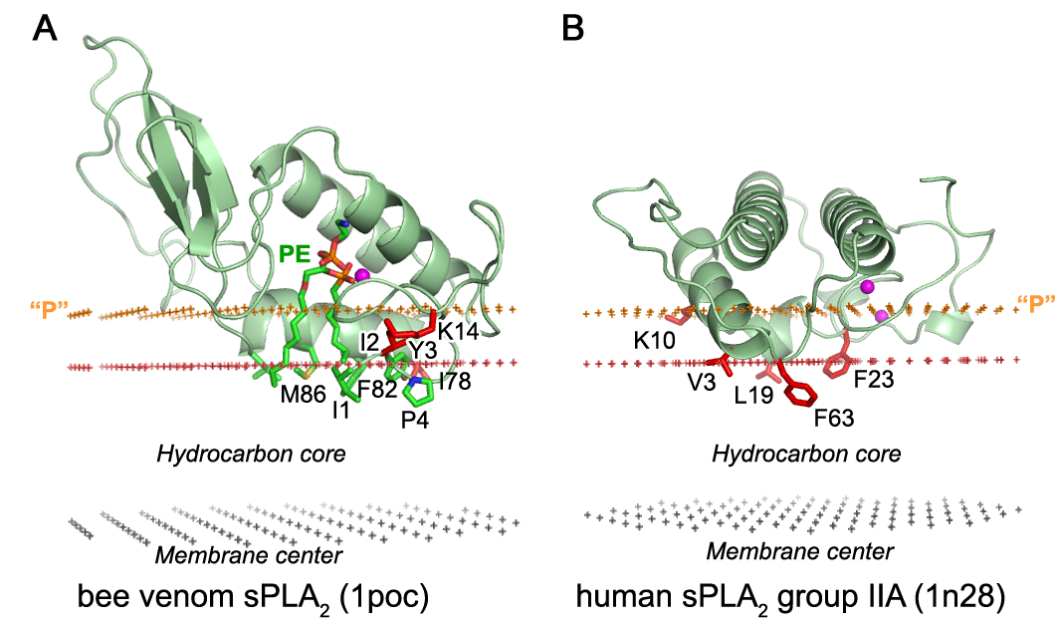
**Calculated membrane binding modes for sPLA_2 _from bee venom with a transition state PE analogue (A) and human group IIA sPLA_2 _(B)**. The backbone of two proteins and the specific lipid ligands are shown in ribbon and stick model, respectively. Residues identified as poorly accessible to polar reagents by SDSL are colored red. Ca^2+ ^ions are shown as balls colored magenta. Hydrocarbon core boundary at the extracellular side is indicated by red dots. The layer of lipid phosphates ("P") is shown by gold dots (at 5 Å outside the hydrocarbon boundary). The center of membrane is indicated by grey dots (at 15 Å inside the boundary).

**Figure 7:**
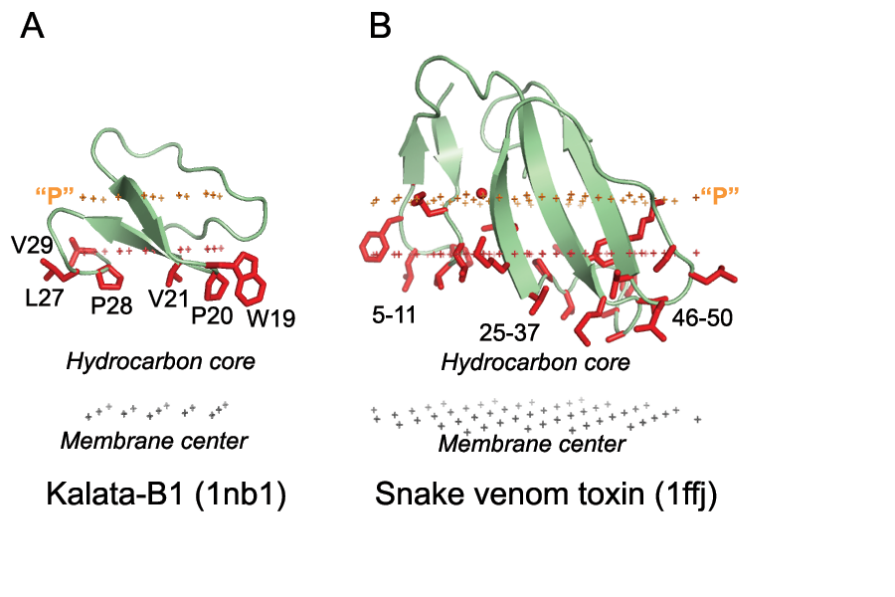
**Calculated membrane binding modes for kalata-B1 plant toxin from cyclotide family (A) and cobra P-type cardiotoxin (B)**. The backbone of two proteins is shown in ribbon model. Micelle-embedded residues identified by solution NMR are colored red. Hydrocarbon core boundary at the extracellular side is indicated by red dots. The layer of lipid phosphates ("P") located 5 Å outside the hydrocarbon boundary is indicated by gold dots, the center of membrane located 15 Å inside the boundary is indicated by grey dots.

To verify the method, we selected a diverse set of 53 peripheral proteins with known 3D structures, whose spatial positions in the membrane have been experimentally studied (Tables 1 and 2, Figures 1, 2, 3, 4, 5, 6, 7; see selection criteria in the Methods). Table 1 includes cases in which individual membrane-bound residues have been determined. These residues either contribute significantly to the membrane binding affinity, as evident from mutagenesis studies, or become inaccessible to water during the protein-membrane association, which can be detected by different physico-chemical methods. Two especially informative methods are site-directed spin labeling (SDSL) and fluorescence quenching, because they allow measuring the membrane penetration depths of individual amino acid residues. Table 2 includes proteins whose overall orientations and membrane-interaction regions have been evaluated by solution or solid-state NMR, fluorescence, or FTIR spectroscopy.

**Table 2:** Predicted penetration depths (*D*) and binding energies (Δ*G*_*calc*_) of peripheral proteins and peptides, whose overall orientations or penetration depths in the lipid bilayer have been experimentally evaluated.

**Protein**	**PDB id**	**Δ*G*_*calc *_(kcal/mol)**	***D *(Å)**	**Method^a^**	**References**
**Lipid clamps**					

C1 domain of protein kinase Cγ	1tbn	-2.3	7.5	NMR	[114]
Annexin 24	1dk5	-1.2	1.8	FL	[115]
Blood coagulation factor VIIa	1dan	-5.7	4.8	FL	[116]
Seminal plasma protein	1h8p	-12.3	9.2	EPR	[117]

**Other proteins**					

Cardiotoxin III	1h0j	-13.1	6.5	FTIR	[118]
Cytotoxin 1	1tgx	-12.4	6.8	FLq	[119]
Cardiotoxin II	1ffj	-18.1	8.7	NMR	[120]
Sapecin	1l4v	-6.6	6.1	NMR	[121]
Cytochrome c	1hrc	-2.0	1.9	SL, NMR	[122, 123]
Coagulation factor IXa	1pfx	-3.8	3.2	FL	[124]
Coagulation factor XIV	1lqv	-4.9	3.5	FL	[125]

**Peptides**					

Alamethicin	1amt	-23.7	28.1	NMR, SL	[44–46]
Zervamicin IIb	1ih9	-14.3	9.9	FL	[47]
Antiamoebin I	1joh	-16.4	14.4	FL	[47]
Magainin	2mag	-14.5	10.1	NMR, FL	[126, 127]
Nisin	1wco	-4.0	8.8	NMR	[128]
Neuropeptide Y	1icy	-9.4	9.4	SL	[129]
Mersacidin	1mqz	-4.9	3.5	NMR	[130]

^a^As in Table 1; FTIR – ATR FTIR spectroscopy.

A comparison with experimental data demonstrates that membrane penetration depths, overall orientations of the proteins, and the sets of their lipid-embedded residues are predicted correctly. The experimentally determined sets of membrane-penetrating residues may be smaller than the sets obtained in calculations (Table 1), mainly because some of the predicted membrane-embedded residues have not been experimentally tested. Importantly, the calculated orientations of homologous proteins in the membrane were always similar, though not identical (Figures 1, 2, 5, 6).

All proteins considered can be separated into two major categories: (1) lipid clamps with cavities or pockets that serve for specific binding of headgroups of certain lipids (Figures 1, 2, 3, 4); and (2) other proteins that interact with lipid bilayers non-specifically (Figures 5, 6, 7). Lipid clamps serve for targeting of proteins to the appropriate cellular membranes rich in the corresponding lipids. For example, binding of PI(4,5)P_2_, PI(3)P, PI(3,5)P_2_, or PI(4)P lipids may target the corresponding domains to the plasma membrane, early endosome, late endosome, or Golgi, respectively [4].

Due to the presence of a few solvent-exposed hydrophobic residues, lipid clamps can be positioned on the membrane surface using our method. However, the binding energies of lipid clamps appear to be small (-2 to -7 kcal/mol, Table 1). This is in agreement with weak affinity of such proteins to artificial membranes in the absence of their cognate lipids, for which the transfer energies are often below the experimental detection threshold of ~3–4 kcal/mol. However, in most cases these hydrophobic interactions were sufficient to properly define the orientations of the proteins, which are in good agreement with known experimental data (see below).

Some lipid clamps have been crystallized with headgroup analogues of their cognate lipids. In these cases acyl chains of the bound lipids were modeled to check whether the overall orientation of proteins may be affected by the presence of bound lipids (see Methods). It appears that calculated spatial positions of these proteins in membranes are usually similar with and without reconstructed lipids. Two exceptions are the EEA1-FYVE dimer and P47^phox^-PX domain, whose orientations are significantly altered by the bound lipids (Figures 2B, 3B). Different orientations of these two proteins in the presence and absence of their cognate lipids are consistent with experimental studies [39,40,88]. Moreover, the orientation of EEA1-FYVE is influenced by its dimerization [10], whereas membrane-protein interactions of P47^phox^-PX domain depend on conformational changes due to protein phosphorylation [38,39].

The majority of peripheral proteins, which have non-polar patches and thus can be treated by our method, do not belong to the lipid clamps category (Tables 1, 2, 3). These proteins are usually attached to the membrane primarily by hydrophobic interactions of exposed non-polar residues (Tables 1 and 2), which penetrate to the hydrocarbon core region. Calculated energies of these proteins are significant (from -5 to -20 kcal/mol; Tables 1, 2, 3). On the other hand, there are cationic proteins that use electrostatics as a means of membrane binding or targeting [4,17-22]. Some of these can still be treated by our method, such as cytochrome c and charybdotoxin from our dataset (Table 4). Some proteins that bind through hydrophobic interactions, such as C2 domain or cPLA_2 _or lipoxigenase, also require Ca^2+ ^binding, which may interact with lipid phosphates [5,34,37] or work as an electrostatic switch [33].

**Table 3:** Predicted penetration depths (*D*) and binding energies (Δ*G*_*calc*_) of peripheral proteins, whose orientations with respect to the membrane have been previously suggested based on their 3D structures

**Protein**	**PDB id**	**Δ*G*_*calc *_(kcal/mol)**	***D *(Å)**	**References**
**Lipid clamps**				

GRK2 kinase -βγ complex	2bcj	-5.5	5.0	[131]
Seminal plasma protein	1h8p	-12.3	9.2	[132]
Myotubularin-related protein	1zvr	-2.3	2.9	[133]

**Other proteins**				

Fatty acid amine hydrolase	1mt5	-30.8	10.0	[134]
Signal peptidase	1kn9	-4.5	4.5	[135]
Lanosterol synthase	1w6k	-19.9	6.5	[136]
Monoamine oxidase	1o5w	-19.9	6.5	[137]
Prostaglandin E synthase	1z9h	-13.1	4.4	[49]
Carnitine O-palmitoyltransferase 2	2h4t	-8.3	3.6	[138]
Major envelope glycoprotein E	1ok8	-9.9	4.9	[139]
Ferrochelatase	1hrk	-9.2	7.2	[140]
Sphingomyelinase C	1zwx	-6.2	6.0	[141]
α-Toxin (bacterial phospholipase C)	1olp	-4.8	3.3	[142]
β-glycosidase	1vff	-6.5	3.3	[143]
Hydroxysteroid dehydrogenase	1y5m	-14.9	3.0	[144]
Carotenoid oxygenase	2biw	-12.7	5.1	[145]
α-Tocopherol transfer protein	1oiz	-20.7	8.4	[146]
Phosphatidylinositol transfer protein	1aua	-14.0	7.8	[147]
Ganglioside GM2 activator	1pub	-7.8	5.2	[148]
Oxysterol-binding protein	1zi7	-5.9	3.0	[149]
Viscotoxin A3	1okh	-4.0	5.8	[150]

^a^Other examples include phospholipases A_2 _and C, microbial and mammalian lipases, annexins, mammalian cytochromes P450, and fatty acid binding proteins [5, 11, 48].

^b^Structures with removed transmembrane helices.

#### Locations of bound lipids, Ca^2+ ^ions, exposed hydrophobic, basic and aromatic residues

**Figure 8:**
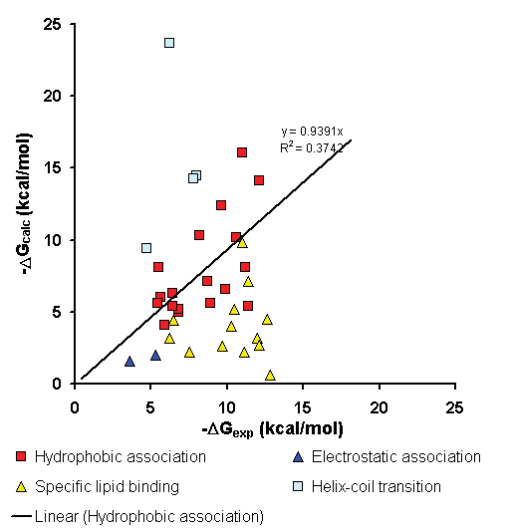
Comparison of calculated (Δ*G*_*calc*_) and experimental (Δ*G*_*exp*_) binding energies for 40 peripheral proteins.

The predicted membrane boundaries are consistent with positions of crystallized lipids in the protein structures. The carbonyl groups of the anchoring lipids are located close to the boundaries of the acyl chain region (blue or red dots in Figures 1, 2, 3, 4, 5, 6, 7, 8), whereas phosphate groups of the bound lipids correspond to the layer of phosphate groups in the surrounding bilayer (gold dots in Figures 1, 2, 3, 4, 5, 6, 7, 8). Thus, the protein-bound anchoring lipids are properly aligned with surrounding fluid lipids, which are not explicitly included in the computational model.

Our calculations are also supported by locations of co-crystallized Ca^2+ ^ions. For example, all bound calcium ions of C2 domains are arranged within 2 Å of the layer of lipid phosphate groups (purple spheres and gold dots in Figure 1, respectively). This allows formation of stabilizing ionic bridges between the Ca^2+ ^ions and phosphate groups of surrounding lipids. One such calcium-bridge can be seen in the crystal structure of PKCα-C2 domain in complex with PS (Figure 1B). According to our results, Ca^2+ ^ions observed in crystal structures of many other proteins, such as phospholipases A2, some lipases, lipoxygenases, α-toxins, and annexin, also appear to be located at the level of lipid phosphates. The possible involvement of Ca^2+ ^ions in interactions with lipid phosphates has been previously discussed [5,33,34,37].

Two other important features are positions of exposed hydrophobic and charged residues of the proteins with respect to the membrane. All exposed non-polar residues and the acyl chains of bound lipids are inserted in the hydrocarbon core of the lipid bilayer (Figures 1, 2, 3, 4, 5, 6). All charged residues are located in the lipid headgroup region outside the calculated hydrocarbon boundaries. A significant part of the positively charged Lys and Arg residues interacts with lipid phosphates at a distance of ~5 Å outside the acyl chain boundaries [28]. Other Lys and Arg residues remain in the aqueous solution at larger distances from the hydrocarbon boundaries and may interact with the negatively charged membrane surface electrostatically or may form ionic pairs with distal phosphates of phosphoinositides (Figures 1).

It is noteworthy that Trp residues of peripheral proteins are frequently involved in the membrane binding (Table 1). According to our results, Trp side chains are usually located at the hydrocarbon boundary, although they can also be found in the membrane interfacial region. Their indole rings are often buried in the hydrocarbon core, while their NH group "snorkels" into the water-saturated interface (Figures 2B, 4A, 5, 7). This provides an additional gain of transfer energy. Such arrangement of Trp residues at the water-hydrocarbon boundary (i.e. at a distances of 15 Å from the membrane center) has been observed in transmembrane proteins [29], and it is energetically preferred in model α-helical peptides [30]. Furthermore, the tryptophan analogue 3-methyl-indole accumulates primarily in the same region of the lipid bilayer [31,32].

#### C2 domains

Spatial positions of twenty different C2 domains with pairwise sequence identities less than 30% were calculated and deposited in OPM. Ten of these proteins were previously included into a dataset of membrane-binding proteins [62]. All C2 domains interact with the membrane via two Ca^2+^- binding loops with exposed hydrophobic residues [33]. Calculated tilts of these domains relative to the membrane normal vary from 20 to 60°.

The spatial positions of four C2 domains in membrane (Figure 1) have been extensively studied using SDSL. This approach allows evaluation of membrane penetration depths of individual spin-labeled Cys residues by measuring their EPR saturation parameters in the presence of non-polar and polar paramagnetic probes, such as molecular oxygen and NiEDa, respectively [34]. The membrane depth parameter (Φ) is determined from the ratio of accessibilities of the nitroxyl label to the non-polar and polar probes. This parameter is equal to zero at the hydrophobic boundary, where the effective concentrations of polar and non-polar probes are approximately equal, and it is positive inside the acyl chain region [6,7,35,36], which is consistent with our previous results for several transmembrane proteins [26].

The comparison with SDSL data indicates that positions of C2 domains are reproduced with reasonable precision. All residues with positive Φ parameters (indicated by red in all Figures) penetrate the acyl chain region (bold in Table 1) or are situated close to calculated hydrocarbon boundaries in the membrane interface (underlined in Table 1). The latter residues are usually polar or charged (as R249 and R250 of PKCα-C2 domain in Figure 1B) and may actually reach the hydrophobic core after their substitution by the non-polar spin-labeled cysteine. The locations of Ca^2+ ^ions (4–6 Å outside the acyl chain region, Figure 1) are consistent with X-ray reflectivity and EPR studies [34,37].

We also found that the C2 domain of cytosolic phospholipase A_2 _(cPLA_2_-C2, 1rlw, Figure 1A) interacts more extensively with the hydrophobic core (ΔG_calc _= -7 kcal/mol) and penetrates the hydrocarbon core by ~2–3 Å deeper than C2-domains of synaptotagmin II (sytI-C2B, 1uov, G_calc _= -4 kcal/mol) and by ~4 Å deeper than the C2 domain of protein kinase Cα (PKCα-C2, 1dsy, ΔG_calc _= -2 kcal/mol) (Figure 1, Table 1). This is consistent with SDSL data [33,34]. The differences in membrane penetration depths correlate with intracellular localizations and lipid binding preferences of corresponding C2 domains. The most hydrophobic and deeply inserted cPLA_2_-C2 domain preferentially interacts with zwitterionic PC-rich membranes, whereas others interact with anionic or PS-rich membranes [4]. For example, PKCα-C2 forms a stable complex with PS as shown in Figure 1B. PS-specific C2 domains are known to interact simultaneously with two types of anionic lipids: they have a binding pocket for PS itself and a cluster of positively charged residues that binds phosphate groups of phosphoinositides (PI) [33]. As shown in Figure 1B, the P4 and P5 atoms of PI are situated at a larger distance from the hydrocarbon boundary (~12 Å) than the P1 phosphate atom of PS (~5 Å). The formation of several ionic bridges with two lipid molecules stabilizes the significant protein tilt with respect to the membrane normal.

#### PX domains

OPM includes five different PX domains, which have similar orientations in lipid-bound form. However, the position of the P47^phox^-PX domain in the membrane is different in the ligand-free conformation, and it is largely regulated by the conformational rearrangement caused by phosophorylation and movement of C-terminal fragment [38,39]. Figure 2 demonstrates spatial positions of two PX domains of NADH oxidase, P40^phox^-PX and P47^phox^-PX, in the lipid-bound conformation. Exposed hydrophobic residues of PX domains, which penetrate into the hydrophobic core in our calculations, were shown to be important for membrane binding (indicated by red in Figure 2, "Bn" data in Table 1).

#### PH domains

Twenty nine different PH domains are currently included in OPM. Among them are eleven domains previously included in a dataset of membrane-binding proteins [62]. Orientation of PLC δ1 PH-domain was identical when calculated with and without bound lipid. Two exposed non-polar residues (V58 and M59) are probably essential for positioning of this domain in the hydrocarbon core region (Figure 3A). Some PH domains from the PDB were not included into OPM, because their calculated energy was close to zero, i.e. calculation did not show any preferential mode of their association with membrane. As was noted before, a large number of PH domains do not bind membranes [20,62]. This may be attributed to several possible reasons: (a) some PH domains probably do not associate with lipid bilayers, but rather interact with other proteins; (b) membrane-interacting loops of PH domains are disordered or missing, especially in NMR models; and (c) the orientation could not be properly determined without the anchoring lipid.

#### FYVE domains

Currently, five different FYVE domains are included in the OPM database. Positions of EEA1-FYVE domains are consistent with data about the importance of hydrophobic residues from their "turret" loop (V1367 and T1368 residues shown in Figure 3B). According to solution NMR studies of the monomeric EEA1-FYVE domain hydrophobic residues from this loop penetrate into the hydrocarbon interior of micelles [10,40]. Surprisingly, the calculated tilt with respect to the membrane plane of monomeric and dimeric FYVE domains differed by ~40° (monomeric 1hyi vs. dimeric 1joc PDB entries), although the same residues from the "turret" loop were buried in the hydrocarbon core in both cases (Figure 3B and 1hyi in Table 1). Thus, the tilt of EEA1-FYVE domain in the membrane is regulated by domain dimerization, in addition to the presence of exposed hydrophobic residues and the specific binding of PI(3)P lipid.

#### C1 domains and ENTH domain of epsin

Some of the membrane-targeting domains have a significant number of exposed hydrophobic residues, such as the previously mentioned cPLA_2α_-C2 (Figure 1A), as well as PKCδ-C1B and epsin-ENTH domains (Figure 4). According to our calculations, C1 domains penetrate deeper into the hydrocarbon core than other membrane-targeting domains, which provides a more significant contribution of hydrophobic interactions (-5 to -6 kcal/mol, Table 1). This is consistent with the binding studies of corresponding C1 domain mutants [89] (shown by red stick in Figure 4).

#### Phospholipases A_2_

Membrane enzymes, such as phospholipases A and C, lipoxygenases, fungal lipases, or cholesterol oxidase, strongly associate with lipid bilayers to extract their hydrophobic substrates (Tables 1, 2, 3). For example, secreted phospholipase A_2 _pulls a phospholipid molecule from the membrane by ~4 Å (Figure 6A). It is generally assumed that the lipid-binding "i-face" of secreted phospholipase A_2 _in the membrane-bound state displaces up to 30 molecules of the surrounding lipids and becomes completely desolvated due to strong hydrophobic interactions with bilayer interior [41]. This is consistent with our results.

The current version of the OPM database includes thirty one secreted phospholipases A_2 _(sPLA_2_). The calculated spatial position of the porcine pancreatic phospholipase, sPLA_2 _group IB (Figure 5A) is quite similar to an arrangement that has been proposed based on the results of fluorescence quenching and ATR FTIR spectroscopy data for a closely related human phospholipase A_2_, whose 3D structure was modeled by homology (Figure 5B) [11]. The results for two other phospholipases are also consistent with experimental data (Figure 6, Table 1). Several lipid-facing residues of these phospholipases were found to be poorly accessible to the polar and non-polar probes. Therefore, it was suggested that these residues possibly face the interfacial region, rather than interact with the hydrocarbon region [42,43]. However, according to our results, these residues pass through the interfacial region and penetrate to the acyl chain region. This is consistent with analysis of fluorescence quenching data and desolvation of i-face of different A_2 _phospholipases [11,41].

#### Proteins that associate non-specifically with lipid bilayers

Many proteins have extensive clusters of exposed non-polar residues that penetrate to the hydrophobic core of the lipid bilayer, according to our results. These proteins include integral monotopic domains, amphiphatic antibacterial peptides, lipopeptide antibiotics (e.g. daptomycin), some polypeptide toxins, water-soluble transporters of small non-polar molecules, and enzymes, such as phospholipases or lipoxygenases (Tables 1, 2, 3).

Calculated membrane penetration depths and orientations of these proteins are consistent with fluorescence quenching, binding, and NMR studies (Figure 7 and Table 1 and 2). For example, the tilt angle of alamethicin with respect to the bilayer normal was estimated as 10–20° [44] while the calculated value was 16 ± 8°. The energetic differences between the transmembrane and surface orientations of all peptaibols (alamethicin, zervamicin, and antiamoebin, Table 2) were found to be < 3 kcal/mol. The transmembrane orientation was energetically preferred for alamethicin and chrysospermin C, whereas the tilted orientation was more favorable for all other peptaibols, in agreement with fluorescence and NMR studies of the peptaibols [44-47].

#### Additional test set of proteins

Orientations of some peripheral proteins in membranes were not investigated directly, but suggested from their crystal structures, presence of acylated residues or other anchoring elements, and indirect biochemical data. These proteins include numerous phospholipases A_2 _and C, microbial and mammalian lipases, annexins, mammalian cytochromes P450, and a wide variety of proteins that transport small non-polar compounds in the cell [5,11,48]. Twenty proteins from this category are included in Table 3. The suggested tentative orientations of all these proteins are consistent with our results, except for the microsomal prostaglandin E synthase [49].

Importantly, the orientation of non-crystallographic symmetry axes in the membrane-bound homo-oligomeric structures may also serve as an internal control. Symmetry axes are usually perpendicular to the membrane, as for example in squalene-hopene cyclase (2sqc), fatty acid amine hydrolase (1mt5), prostaglandin E synthase (1z9h), corticosteroid dehydrogenase (1y5m). However, a deviation of ~3° from the perpendicular direction was observed for the prostaglandin H2 synthase dimer, since the structures of the protomers are not completely identical. The symmetry axis of monoamine oxidase dimer was tilted even more (by ~8°) with respect to the normal, probably because this enzyme works as a monomer, and the hydrocarbon boundary of the crystallized dimer was poorly approximated by a plane.

### 2. Comparison with experimental membrane-binding free energies

An important question is whether the calculated protein-membrane binding energies are reasonable. These energies can be compared with experimental membrane binding affinities determined for a number of proteins with known 3D structures (Table 4). All these proteins were separated into several categories depending on their tentative membrane binding mechanisms: (1) nonspecific hydrophobic association; (2) lipid clamps; (3) mostly electrostatic association based on the ionic strength dependence of their membrane binding affinities; and (4) peptides that undergo helix-coil transitions upon association with membranes.

**Table 4:** Comparison of calculated binding free energies (ΔG_calc_) and maximal experimental (ΔG_exp_) binding free energies (kcal/mol) to lipid bilayer for different peripheral proteins.

**Protein name**	**PDB id**	**ΔG_calc _(kcal/mol)**	**ΔG_exp _(kcal/mol)^a^**	**Reference**
**Predominantly nonspecific hydrophobic association**				

Cholesterol oxidase (*Brevibacterium*)	1coy	-4.1	-5.9	[105]
Cholesterol oxidase (*Streptomyces*)	1b4v	-7.1	-8.7	[151]
8R-lipoxygenase	1zq4	-5.0	-6.8	[104]
Snake phospholipase A_2_, group I	1poa	-5.4	-11.4	[98]
Snake phospholipase A_2_, group II	1vap	-10.2	-10.6^c^	[98]
Human phospholipase A_2_, group II	1n28	-6.3	-6.4	[57]
Voltage sensor toxin	1s6x	-5.2	-6.8	[152]
Kalata B1	1nb1	-5.4	-6.4	[153]
Phospolipase C	2ptd	-6.0	-5.6	[101]
Insect phospholipase A_2_	1poc	-10.3	-8.2	[154]
Human phospholipase A_2_, group X	1le6	-21.8	-6.1	[57]
Octreotide -2	1soc	-5.6	-5.4	[155]
Pancreatic lipase	1ethA	-16.1	-11.0	[156]
Cytotoxin 1	1tgx	-12.4	-9.6	[119]
Sapecin	1l4v	-6.6	-9.9	[121]
C1 domain of Raf-1 kinase	1faq	-8.1	-5.5	[2]
Gramicidin S	1tk2	-14.1	-12.1	[157]

**Specific binding of lipid ligands (lipid clamps)**				

C2 domain of cPLA_2_	1rlw	-7.1	-11.4^c^	[84]
cPLA_2 _holoenzyme	1cjy	-9.8	-11.0	[158]
Phospholipase Cδ1	1djx	-3.2	-12.0^b^	[159]
C2 domain of synaptotagmin IA	1byn	-4.4	-6.5	[160]
C2 domain of PKCβ	1a25	-2.2	-7.5	[160]
C2 domain of PKCα	1dsy	-0.6	-12.8^c^	[161]
C2 domain of PTEN	1d5r	-2.6	-9.7	[162]
C2 domain coagulation factor Va	1sdd	-5.6	-8.9^c^	[163]
C2 domain coagulation factor VIII	1d7p	-8.1	-11.2	[92]
FYVE domain of Vps27	1vfy	-4.0	-10.3	[164]
ENTH domain of epsin	1h0a	-5.2	-10.5	[77]
PX domain (p40phox)	1h6h	-4.5	-12.6	[39]
PX domain (p47phox)	1o7k	-2.7	-12.1	[39]
PH domain of PLC-δ1	1mai	-3.2	-6.2	[165]
Equinatoxin II	1iaz	-2.2	-11.1	[166]

**Predominantely nonspecific electrostatic binding**				

Charybdotoxin	2crd	-1.6	-3.6	[17]
Mitochondrial cytochrome c	1hrc	-2.0	-5.3	[76, 167]

**Helix-coil transitions during binding**				

Magainin	2mag	-14.5	-8.0	[168]
Alamethicin	1amt	-23.7	-6.2	[169]
Zervamicin	1ih9	-14.3	-7.8	[47]
Neuropeptide Y	1icy	-9.4	-4.7	[125]

^**a **^Experimental values were taken for lipid compositions that provided maximal binding affinity and at the ionic strength close to physiological conditions (~0.1 M of KCl). *ΔG*_*exp *_values were taken from the original publications or calculated from the published molar partition coefficients of the proteins as -RT lnK_*d*_.

^b ^Measured for isolated C2 domain of the protein.

^c ^Different binding affinities were determined for these proteins in other studies [8, 160, 170, 171].

The free energy of protein-membrane association (*ΔG*_*bind*_) includes several components [50,51]:

Δ*G*_*bind *_= Δ*G*_*transf *_+ Δ*G*_*head-group *_+ Δ*G*_*spec *_+ Δ *G*_*pKa *_+ Δ *G*_*conf *_+ Δ*G*_*bilayer *_+ Δ*G*_*imm*_

where *ΔG*_*transf *_is transfer energy of protein atoms from water into the hydrocarbon interior of the membrane; *ΔG*_*head*-*group *_describes electrostatic, H-bonding and other "non-specific" interactions of the protein with headgroups of fluid lipids; *ΔG*_*spec *_is "specific" binding energy of the lipids, which are inserted as ligands into the protein cavities; *ΔG*_*pKa *_is an ionization energy of charged groups that lose their charges when transferred from water into the non-polar environment; *ΔG*_*conf *_represents changes in thermodynamic stability of the protein during its insertion into the membrane; *ΔG*_*bilayer *_is a deformation energy of the lipid bilayer that appears due to non-zero lateral pressure or hydrophobic mismatch; and *ΔG*_*imm *_is an immobilization free energy of the protein. The first three terms in this equation usually stabilize the protein-membrane association, while the last four are mostly destabilizing, although the contribution of the lateral pressure ("intrinsic curvature") can be positive or negative [52].

Experimental binding energies of peripheral proteins depend on the specific lipid composition of the membrane [53]. For example, the presence of negatively charged lipids improves the binding of many peripheral proteins. This effect can be attributed to a variety of reasons, including electrostatic attraction of cationic proteins to negatively charged lipids (*ΔG*_*head*-*group*_), specific binding of anionic lipids to protein cavities (*ΔG*_*spec*_), or reduced lateral pressure and increased hydration in the membrane interfacial region (*ΔG*_*bilayer*_) [51]. For the sake of comparison, we selected only *maximal *experimental membrane binding affinities of the proteins, which were measured under the lipid compositions most favorable for binding.

Our computational approach includes only transfer and ionization energy contributions (*ΔG*_*transf *_and *ΔG*_*pKa *_in equation (2)), which are independent of the lipid composition. The hydrocarbon interior of the membrane was treated essentially as non-polar liquid, with interfacial polarity profiles derived from EPR studies. In this approximation, protein-membrane binding is driven by hydrophobic interactions and opposed by desolvation of polar groups and deionization of charged residues. All other energy contributions were temporarily neglected, since they are strongly dependent on the lipid composition (*ΔG*_*head*-*group*_, *ΔG*_*spec*_, and *ΔG*_*bilayer*_). Therefore, some discrepancies are unavoidable.

Nevertheless, for the majority of proteins from group (1), calculated and experimental energies correlate for proteins from the first group (red squares in Figure 8). The observed differences between calculated and experimental binding energies were relatively small, from 1 to 3 kcal/mol. Thus, in these cases, the non-specific hydrophobic interactions probably account for ~50–90% of experimental binding energies (Table 4). The neglected contributions are either relatively small or cancel each other.

However, the correlation does not hold for other groups of proteins (Figure 8). The energies were strongly underestimated for membrane targeting domains (PH, PX, FYVE, C2) and equinatoxin, which are known to associate specifically with certain types of lipids. Calculated energies of these lipid clamps differed by ~5–9 kcal/mol from experimental energies measured in the presence of specifically bound lipids (Figure 8). This was expected, because the affinities of these proteins to membranes are weak in the absence of anchoring lipids. Thus, the omitted specific binding energy with headgroups of lipids (*ΔG*_*spec*_) appeared to be predominant (up to 9 kcal/mol) for these proteins.

Calculated energies were also underestimated for cytochrome *c *and charybdotoxin whose binding is known to depend on electrostatic interactions (a part of the omitted *ΔG*_*head*-*group *_term). However, the electrostatic energy was relatively small, ~3 kcal/mol judging from the deviations in Figure 8 (all experimental data were taken for physiological concentrations of ions, usually ~0.1 M KCl).

In contrast, calculated energies were overestimated for peptides that undergo helix-coil transitions during their binding to the membrane, such as magainin and peptaibols. The energies were calculated for α-helices that are found in crystals or in micelles, though such peptides are unfolded in aqueous solution. The energetic costs associated with folding of the α-helices from coil can be significant, because they represent a combination of backbone energy (which is close to zero at 300 K) and a sum of α-helical propensities of all residues in the helix [54,55]. The propensities are positive (destabilizing) and vary from zero to ~1 kcal/mol for individual residues, and up to 4 kcal/mol for proline. Thus, *ΔG*_*conf *_may be large for peptides or proteins that undergo significant conformational changes during membrane binding, such as, lipases, or channel-forming toxins.

The calculated free energy was also strongly overestimated for phospholipase A_2 _from group X that has an unusually large exposed hydrophobic surface (1le6, not shown in Figure 8). It has been demonstrated that this protein easily associates with zwitterionic lipids at concentrations lower than critical micelle concentration [56]. Therefore, the experimental data [57] may reflect membrane binding affinity of a preexisting enzyme-lipid complex (that has a small exposed non-polar surface) rather than of a lipid-free enzyme.

This analysis shows that the most significant energetic contributions to binding energy for some proteins come from their transfer energy *ΔG*_*trans*_, specific binding of lipid ligands *ΔG*_*spec *_(for lipid clamps), and changes of protein stability *ΔG*_*conf *_(for peptides that undergo helix-coil transitions). Electrostatic interactions are less significant, although essential for binding of cationic proteins. Ionization energy is usually small because all ionizable groups of a typical peripheral protein remain outside the hydrophobic slab after energy minimization. The omitted membrane deformation energy, which depends on lipid composition, is also relatively small: it has been evaluated as ~2–4 kcal/mol for α-helical peptides [58]. Free energy of immobilization was estimated as only ~1kcal/mol [27]. This explains the relatively small discrepancies in the energies for proteins from set (1) (Figure 8).

### 3. Main categories of membrane-associated proteins

After initial testing, the method was applied for identification and characterization of a wide spectrum of membrane-associated protein structures from the PDB. These structures were divided into three groups: (A) peripheral domains of integral transmembrane proteins; (B) integral monotopic proteins that are permanently membrane-associated; and (C) peripheral proteins that exist in free and membrane-bound states (see Table 5 and Additional file 1). Most of the selected proteins probably interact with lipid bilayers *in vivo*, as follows from UniProt and PubMed records, although some of them can only be tentatively assigned as membrane-associated. Membrane-interacting domains belong to 126 different superfamilies and 173 families based on SCOP classification [59]. Calculated transfer energies of these structures ranged from -2 to -38 kcal/mol, and membrane core penetration depths were between 1 to 15 Å (Table 5). The results are less reliable and accurate for proteins with small transfer energies: the fluctuations of their penetration depths and tilt angles reached 3 Å and 20°, respectively, within the energy interval of 1 kcal/mol. These fluctuations are larger than for transmembrane proteins (up to ± 1.5 Å and ± 5°, respectively, [26]).

**Table 5:** Typical membrane binding elements, calculated energies (Δ*G*_*calc*_, kcal/mol) and membrane penetration depths (D, Å) for different categories of membrane-associated proteins (see Additional file 1)

**Category**	**N^a^**	**Binding elements**	**Δ*G*_*calc *_(kcal/mol)**	***D***
A. Extramembrane domains of transmembrane proteins	29	α-helices, loops	-2.4 to -30.8	2.3 to 12.5
B. Integral monotopic	6	α helices,	-8.6 to -38.3	4 to 10
C.1. Peripheral enzymes	119	α-helices, loops	-1.4 to -38.2	1 to 13
C.2. Water-soluble carriers of non-polar substances	42	α-helices, loops, β-sheets	-2.2 to -20.7	2 to 8
C.3. Membrane-targeting and other structural domains	93	loops, α-helices, bound lipids	-1.2 to -12.3	1 to 9
C.4. Electron carriers	24	Loops	-1.2 to -7.5	1 to 5
C.5. Polypeptide ligands (hormones, toxins, inhibitors)	101	β-sheets, α-helices, loops	-1.6 to -18.1	1 to 18
C.6. Water-soluble forms of channel-forming polypeptides	38	α-helices, loops	-1.3 to -20.9	1 to 28

^**a**^Number of different protein structures in the set.

Peripheral domains of transmembrane proteins (group "A") are usually water-soluble. However, some of them require detergents for extraction or crystallization, even after removal of their hydrophobic transmembrane α-helices. Therefore, such domains are often described as integral monotopic [60]. Among them are monoamine oxidases A and B, fatty acid amide hydrolase, mammalian cytochromes P450, corticosteroid dehydrogenases, and major envelope glycoprotein.

True integral monotopic proteins (group "B") do not have membrane-spanning α-helices, by definition. Six integral monotopic proteins from the PDB include prostaglandin H2 synthases 1 and 2 (1q4g and1cx2), lanosterol synthase (1w6k), squalene-hopene cyclase 2sqc), microsomal prostaglandin E synthase (1z9h), and carnitine O-palmitoyltransferase 2 (2h4t).

All peripheral proteins (group "C") are water-soluble, a least in one of their conformational states [2]. These proteins usually reversibly associate with lipid bilayers. However, some polypeptide toxins can undergo conformational transitions and form transmembrane channels that are irreversibly associated with membranes, as in the case of α-hemolysin. Some other water-soluble proteins may also adopt a transmembrane orientation during intermediate steps of their macromolecular assembly, as pilin IV, which forms the bacterial pilus [61], and the major coat proteins of filamentous phages. Such structures are not considered here but are included in OPM. Peripheral proteins were divided into six functional categories (C1-C6 in Table 5) that differ in typical membrane-anchoring motifs, strength of hydrophobic interactions with lipid bilayers, and membrane penetration depth.

The first category (C.1.) includes 102 enzymes that participate in metabolism of different membrane components, such as lipids (e.g. phospholipases and cholesterol oxidases), cell wall oligosaccharides (e.g. glycosyltransferase and transglycosidases), or proteins (e.g. signal peptidase and palmitoyl protein thioesterases). They also process some hydrophobic substrates that can be dissolved in the membranes (e.g. substrates of carotenoid oxygenase) or exist as lipid micelles or non-polar droplets (e.g. substrates of pancreatic lipases). Calculated energies and penetration depths of many enzymes are relatively small either because the crystallized proteins were in the "closed state", less favorable for membrane binding (Table 6), or because some of their membrane-anchoring α-helices or loops were disordered or missing in the crystal structures.

**Table 6:** Calculated parameters of open and closed states of membrane-associated proteins: binding energies (Δ*G*_*calc*_, kcal/mol), penetration depths (*D*, Å), and tilt angles (τ, °).

**Protein**	**Category**	**"Closed" state**				**"Open"/lipid-bound state**			
		
		**PDB id**	**Δ*G*_*calc*_**	***D***	**τ**	**PDB id**	**Δ*G*_*calc*_**	***D***	**τ**
Cytochrome P450 2b4	A.1	1suo	-13.0	4.7	54	2bdm	-18.2	10.5	45
Fungal lipase 1	C.1	1trh	-7.8	3.0	80	1lpp	-30.6	9.1	84
Triacylglycerol lipase *Rhizomucor miehei*	C.1	3tgl	-4.1	4.5	28	4tgl	-14.0	4.9	55
Triacylglycerol lipase *Humicola lanuginose*	C.1	1tib	-3.3	1.9	42	1ein	-13.5	9.5	71
Gastric lipase^a^	C.1	1hlg	-5.0	3.4	6	1k8q	-10.8	6.3	80
α-Toxin (Phospholipase C)	C.1	1gyg	-4.2	5.5	60	1ca1	-4.5	2.2	88
Alpha-tocopherol transfer protein	C.2	1r5l	-10.6	4.0	81	1oiz	-20.7	8.4	63
Glycolipid transfer protein	C.2	1swx	-4.9	3.4	86	1sx6	-7.9	3.9	87
Ganglioside GM2 activator	C.2	2ag4	-5.1	4.2	47	1tjj	-9.3	4.6	55
Synaptotagmin C2A domain of ^b^	C.4	1rsy	-3.3	2.1	26	1byn^c^	-4.4	3.7	36
Mersacidin^d^	C.7	1mqx^c^	-2.3	2.4	77	1mqy^c^	-9.5	11.5	56

^a ^The open and closed forms of gastric lipase are taken from closely related species.

^b ^Ca^2+^-free structure (1rsy) versus Ca^2+^-bound structure (1byn). Ca^2+ ^-bound state is usually referred as associated with the lipid bilayer.

^c ^Structures determined by solution NMR spectroscopy. All other structures were solved by X-ray crystallography.

^d ^"Closed" – structure in methanol solution, "open" – structure in DPC micelles. Crystal structure of the peptide (1qow) has intermediate values of Δ*G*_*calc *_= -7.9 kcal/mol and *D *= 6.9A (in the monomeric state).

The second category (C.2.) includes 42 carriers that transfer small non-polar compounds between different types of cell membranes or between membranes and cytosolic protein complexes. The transported substances include phosphatidylinositol, α-tocopherol, gangliosides, glycolipids, sterol derivatives, retinol and fatty acids.

The third category (C.3.) includes 85 membrane-targeting and other structural domains that mediate attachment of other proteins to membranes and may be involved in subcellular targeting and signal transduction [4,62]. These domains are usually attached to the membranes by the specific non-covalent binding of their cognate phospholipids (PE, PS, PIPs). However, non-specific hydrophobic and electrostatic interactions also play an important role. The interactions with lipids are also mediated by Ca^2+ ^ions. Therefore, the presence of Ca^2+ ^or specific phospholipids targets them to specific cellular compartments.

The fourth and fifth categories include 24 electron carriers (C.4.) and 88 different polypeptide ligands, i.e. hormones, toxins, inhibitors, or antimicrobial peptides (C.5). These proteins interact specifically with large transmembrane proteins. However, they may also be accumulated at the membrane surface prior to binding their protein targets. The carriers and polypeptide ligands are often positively charged and interact electrostatically with anionic membranes. The hydrophobic interactions of such proteins with membranes can vary from small to very significant.

The final category (C.6) includes 38 channel-forming polypeptides that undergo oligomerization and significant conformational transitions and thus may associate with membranes irreversibly. The structure of the membrane-bound state has been determined only for α-hemolysin. In all other cases, the experimental structure represents a water-soluble conformation that only weakly binds to the lipid bilayer. It is noteworthy that such proteins are usually present as monomers in the crystals, although they form oligomers in membranes. Only alamethicin, mersacidin, tsushimycin, and one of δ-endotoxins form dimers or trimers in crystals that might be biologically relevant.

## Discussion

Orientations of many proteins in membranes have been experimentally studied (Tables 1 and 2), suggested from their crystal structures (Table 3), or theoretically predicted (a few dozens of cases). However, the coordinates of proteins with membrane boundaries are publicly accessible only for a homology model of human pancreatic phospholipase A_2 _([11], Figure 5b) and several cardiotoxins available from authors upon request. Calculated spatial positions of other proteins in membranes can only be roughly estimated from the published pictures.

In the present work, we calculated the positions in membranes for more than 470 membrane-associated proteins and peptides, compared the results with available experimental data, and deposited all coordinates of proteins oriented in the lipid bilayer in our OPM database for easy public access [24]. At the present time, OPM is the only database that provides positions of peripheral and integral membrane proteins of known 3D structure in the lipid bilayer along with their structural classification, oligomeric states, topologies and subcellular localizations. Other bioinformatics resources focus only on transmembrane and a few integral monotopic proteins [63-65].

### 1. Applicability of the method

The large-scale computational analysis was accomplished using the hydrophobic slab model of the lipid bilayer implemented previously in our program PPM 1.0 [26]. In this model, protein-membrane association is driven by hydrophobic interactions that provide negative transfer energy. An opposite destabilizing contribution comes from the desolvation of polar and ionizable protein groups (equations (2–5)). Long-range Coulomb electrostatic interactions of the protein with headgroups of lipids were not included, because they strongly depend on specific lipid compositions of different biological membranes. This approach was previously verified only for transmembrane proteins, and for these systems was shown to be more consistent with experimental data [26] than other computational approaches, such as TMDET [63] or IMPALA [66].

Peripheral proteins represent a significant challenge for this method, because they have relatively small exposed non-polar regions and their hydrophobic interactions with lipid bilayers might be overridden by electrostatic or other interactions with headgroups of lipids, unless they work in concert with hydrophobic forces. In spite of potential complications, we found that PPM 1.0 performed surprisingly well, since the results were in close agreement with experimental data for the test set of 53 well-studied peripheral proteins and peptides (Tables 1, 2, 3, Figures 1, 2, 3, 4, 5, 6, 7). The method was applicable due to the presence of exposed non-polar patches at the surfaces of all peripheral proteins in the dataset, which is sufficient for positioning of proteins in the lipid bilayer.

Our primary goal was to reproduce the spatial positions of proteins in membranes, rather than their binding affinities. However, the calculated transfer energies were fairly consistent with experimental binding energies for many peripheral proteins (Figure 8). The discrepancies of binding energies (but not the calculated orientations) have only been observed in special cases that involve specific lipid binding, helix-coil transitions, or predominantly electrostatic associations (Figure 8, Table 4).

Due to its relative simplicity, our method can be applied to high-throughput screening of proteins with exposed non-polar patches that can potentially associate with membranes, unlike more complex and computationally expensive molecular dynamics simulations. The orientations obtained for numerous peripheral proteins from the PDB are in line with expectations of crystallographers (Table 3) and consistent with the arrangement of detergents, lipids, non-polar binding cavities, acylated residues and other structural features indicating which regions of the proteins are involved in membrane associations (see Results). Unfortunately, fully automated detection of membrane-associated proteins was not possible due to several limitations. First, many structures of peripheral proteins in the PDB are incomplete, because some or all of their anchoring elements are disordered or removed for crystallization. Second, some crystal or solution structures are different from the membrane-bound conformations of the corresponding protein. Third, it is often important to know a complete quaternary structure of a protein complex, rather than the structure of an individual polypeptide chain or a domain. Finally, it was essential to check the biological relevance of the detected protein-membrane association modes based on the literature and UniProt records, but such information is not always readily available or is difficult to interpret.

Apparently, our version of the continuum solvent approach can also be applied for positioning homology models of peripheral proteins in membranes, in addition to the experimental structures as in the present study. The method can be further improved by including free energy of helix-coil transitions for unfolded peptides [55,67] and energetic contributions that are dependent on the lipid compositions of membranes, such as Coulomb electrostatic interactions, lateral pressure [53], and the hydrophobic mismatch [68].

### 2. Importance of hydrophobic interactions with the bilayer core

It is generally accepted that protein-membrane binding is driven by a combination of hydrophobic, electrostatic and other interactions (equation (1)). However, the model applied here includes only hydrophobic interactions, desolvation energy of polar groups, and ionization energy. This model was tested for two different datasets: (a) proteins whose spatial positions in the lipid bilayers or membrane binding affinities have been experimentally quantified *in vitro *and (b) a set identified from screening the PDB and subsequent analysis of results and relevant data and literature. The former set is not biased, since all appropriate examples were simply selected from the literature (see Methods). The latter set may be biased toward proteins that are more amendable to our method. However, this second set was extremely diverse. It included 6 integral monotopic, 415 peripheral proteins, and 55 membrane-associated peptides from the PDB, which were classified into seven functional categories: enzymes, structural and regulatory domains, membrane-targeting domains (lipid clamps), transporters of hydrophobic substances, electron carriers, polypeptide ligands (hormones, inhibitors, toxins, and antimicrobial peptides), and channel-forming polypeptides (Table 5).

The ability of the method to accurately predict the positions and orientations of hundreds of peripheral proteins indicates the importance of hydrophobic interactions for protein-membrane association. All these proteins, including lipid clamps, have some surface non-polar residues that associate with the bilayer core. These residues were sufficient for defining a unique spatial position of the proteins even in the cases in which the binding is driven by specific protein-lipid interactions.

The important role of hydrophobic interactions is expected. Peripheral proteins are known to associate with lipid bilayers through various hydrophobic anchors, such as amphiphilic α-helixes, exposed non-polar loops, or acylated amino acid residues [2]. Hydrophobic interactions of non-polar or acylated residues are essential even for highly cationic peptides and proteins of natural origin, such as the polybasic domain of MARCKS protein or the pH sensor hisactophilin [18,69]. It has been shown that even unfolded peptides may penetrate through the lipid headgroup region and reach the hydrocarbon interior of the membrane if they have a few non-polar residues [70-72], similar to amphiphilic α-helical peptides [73,74].

A variety of typical membrane-anchoring structures was found during our computational analysis of the large set of diverse proteins. For example, amphiphilic α-helices serve as membrane anchors in all integral monotopic proteins, in a majority of membrane-associated enzymes, and in many channel-forming peptides (Table 5). On the other hand, snake venom toxins, defensins, and some antimicrobial peptides may interact with membranes through amphiphilic β-hairpins, β-sheets or β-turns. However, an exposed hydrophobic loop likely represents the most common structural element that may either interact with the membrane interface or penetrate into the hydrophobic region. A typical membrane-bound hydrophobic loop looks like an elongated protrusion that penetrates through the interfacial regions of the lipid bilayer. Such protrusions usually present some exposed hydrophobic (often aromatic) residues at their tips, flanked by basic and aromatic residues (Lys, Arg, Tyr, Trp) that interact favorably with headgroups of lipids [75]. The irregular shape of membrane-binding loops facilitates the creation of binding cavities for lipids that specifically interact with headgroups of lipids, such as annexins or anemone cytolysins.

**Figure 9:**
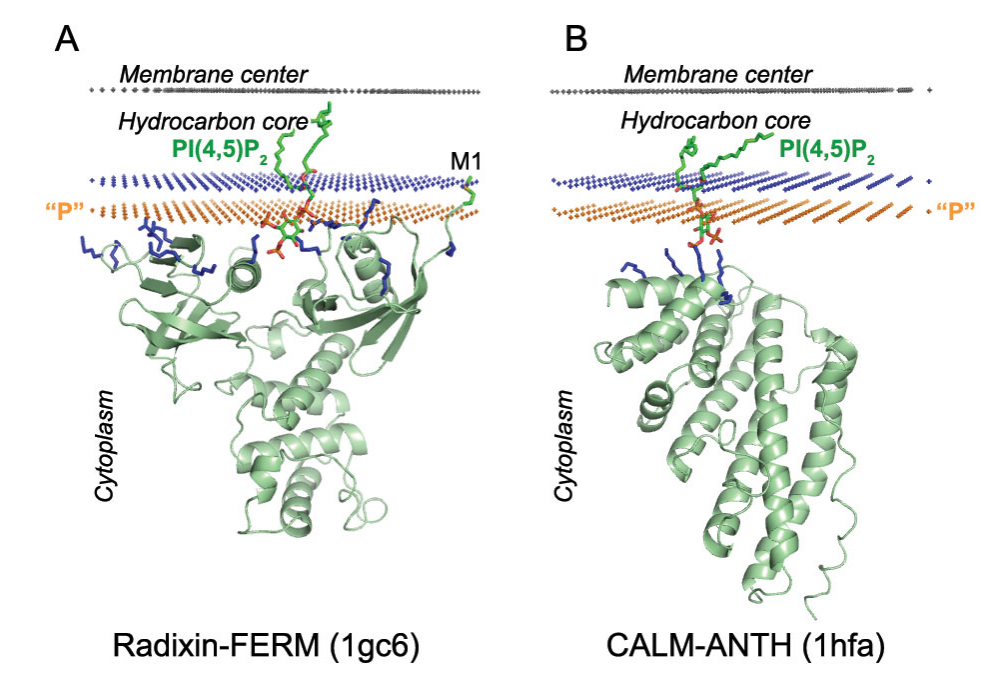
**Calculated membrane binding modes for Radixin-FERM domains with lipid ligand PI(4,5)P_2 _(A) and CALM-ANTH with lipid ligand PI(4,5)P_2 _(B)**. The backbone of two S-type domains and the specific lipid ligands are shown in ribbon and stick model, respectively. Cationic residues involved in ligand and membrane interactions are colored blue. Hydrocarbon core boundary at the cytoplasmic side is indicated by blue dots. The layer of lipid phosphates ("P") is shown by gold dots (at 5 Å outside the hydrocarbon boundary). The center of membrane is indicated by grey dots (at 15 Å inside the boundary).

The hydrophobic interactions are not necessarily predominant. Another very important and common mechanism is the specific non-covalent binding of regulatory lipids, as has been found for membrane-targeting domains [4]. Non-specific electrostatic interactions are also present and may be important for targeting of the proteins to their destination membranes. Electrostatic interactions are relatively weak at physiological ionic strength. They account for 3 to 4 kcal/mol for small cationic proteins, such as cytochrome c, charybdotoxin (Figure 8), or hisactophilin [17,67,76], interacting with negatively charged membranes. It is noteworthy that such interactions are probably not long-range, but rather involve formation of ion pairs, especially between clusters of basic residues and phosphoinositides (Figures 1B and 9).

The results of our calculations are consistent with classification of peripheral proteins as H, I, and S-types [4]. H-type proteins and peptides have many exposed non-polar residues that penetrate into the hydrocarbon core region. They include integral monotopic domains, amphiphatic antibacterial peptides, lipopeptide antibiotics (e.g. daptomycin), some polypeptide toxins, water-soluble transporters of small non-polar molecules, such enzymes as phospholipases and lipoxygenases, and probably several membrane-targeting domains (cPLA_2_-C2, PKCδ-C1B, Epsin-ENTH).

I-type proteins have fewer exposed non-polar residues. They may only contact with the hydrocarbon core by their non-polar residues but stay primarily in the membrane interfacial region. A majority of membrane-targeting domains, such as C2, PX and PH, discoidin domains (Figures 1, 2, 3) and annexins, belong to this category.

S-type ("surface") proteins are usually cationic, have only one or two exposed non-polar residues, and bind their anchoring lipids with relatively low affinity and specificity. Association of S-proteins with membranes strongly depends on the ionic strength [77]. These proteins are usually described as involved in long-range electrostatic interactions with the membrane surface, while remaining in the aqueous solution [17]. However, according to our results, Lys and Arg residues of these proteins may penetrate into the interfacial region and form ionic pairs with lipid phosphate groups (as in radixin-FERM, Figure 9A). Moreover, even proteins that are located at the membrane surface (as CALM-ANTH in Figure 9B) may also form direct ionic bridges with P4 and P5 of phosphoinositides in addition to the long-range electrostatic interactions with the membrane surface. Such protein-lipid ionic bridges may be weakened at high ionic strength [77], just as ionic pairs in peptides [78].

### 3. Conformational changes during protein-membrane association

Typical peripheral proteins are prone to conformational changes in response to phosphorylation or binding of ions, ligands, or other proteins [5]. Structural changes range from rearrangements of side chains and loops to refolding and significant movements of regular secondary structures. Such changes may promote the protein binding to the membrane, which in turn stabilizes the membrane-bound conformation [2]. Some examples of conformational rearrangements are shown in Tables 6 and 7.

**Table 7:** Comparison of calculated energies (Δ*G*_*calc*_, kcal/mol), predicted penetration depths (*D*, Å) and protein tilts with respect to the membrane normal (τ, °) for alternative conformations of membrane-associated loops of different peripheral proteins.

**Protein**	**Category**	**Conformation 1^a^**				**Conformation 2^a^**			
		
		**PDB id**	**Δ*G*_*calc*_**	***D***	**τ**	**PDB id**	**Δ*G*_*calc*_**	***D***	**τ**
Signal peptidase	A.1	1kn9	-4.5	4.5	83	1t7d	-5.9	3.7	66
Cytochrome P450 2b4	A.1	1po5	-6.9	9.7	39	2bdm	-18.2	10.5	45
Cytochrome P450 2c5	A.1	1nr6	-11.6	12.5	54	1dt6	-8.4	7.5	17
Cytochrome P450 2c9	A.1	1og5	-13.6	7.5	74	1r9o	-10.7	7.5	57
Cytochrome P450 3a4	A.1	1tqn	-20.7	10.2	61	1w0f	-15.2	6.2	68
Bile-salt activated lipase	C.1	1aql	-9.7	6.0	80	1akn	-6.6	5.7	67
Triacylglycerol lipase	C.1	1tib	-3.3	1.9	42	1dt5	-4.8	2.9	84
Lipase/colipase complex	C.1	1lpa	-22.1	8.7	89	1lpb	-26.2	10.3	87
Ganglioside GM2 activator	C.2	1tjj	-9.3	4.6	55	1pub	-7.8	5.2	44
Phosducin/βγ complex	C.3	1a0r	-3.7	2.7	45	2trc	-2.9	4.4	64
Tubby protein	C.4	1c8z	-4.2	6.2	87	1i7e	-3.6	2.6	78

^**a **^All structures were solved by X-ray crystallography.

The majority of experimental structures represent the "closed" state, which is more stable in aqueous solution. Relatively few structures represent an "open" state that is more favorable in membranes (Table 6). During crystallization, "open" states can be stabilized by lipids (lipases, glycolipid transfer protein, or ganglioside GM activator), detergents (α-tocopherol transfer protein), micelles (mersacidin), pH changes (α-toxin), Ca^2+ ^ions (C2A domain of synaptotagmin), or by formation of presumably non-native dimers (one of cytochromes P450).

According to our results, calculated energies of proteins in the "open states" are usually lower than in the "closed states" (Table 6). Thus, "open" conformations are more prone to membrane association. Further, the "open" conformation also penetrates deeper into the membrane. Predicted membrane binding regions are overlapped in the different states of the proteins, although they may slightly differ. The initial weak association of the "closed" state to the membrane facilitates its subsequent transformation to the "open" (productive) state. On the other hand, the conformational change from the "open" to the "closed" state may be required for dissociation of the protein from the membrane.

There are also many cases in which the alternative structural states are not defined as "closed" and "open", although they have different conformations of membrane-interacting loops due to ligand binding, different crystallization conditions, or cleavage of different segments of the polypeptide chain. The calculated spatial positions of such conformational states in the membrane can also be somewhat variable (Table 7).

### 4. Comparison with other computational methods

The positions of proteins in membranes can be simulated using three different computational approaches: (a) energy minimization using the hydrophobic slab approximation of the lipid bilayer (as in the present study), (b) molecular dynamics (MD) simulations with explicit lipids, or (c) optimization of Coulomb electrostatic interaction energy of the protein with a charged planar membrane surface.

**Figure 10:**
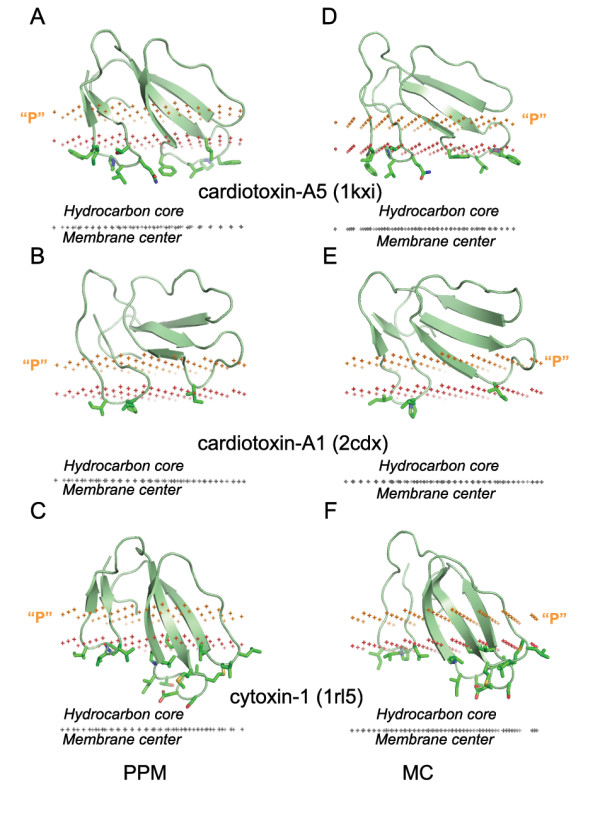
**Comparison of positions in the lipid bilayer of homologous toxins calculated by different methods**. Cardiotoxin A5 (A, D), cardiotoxin A1 (B, E) and cytoxin 1 (C, F) were calculated byPPM (A-C) or by Monte Carlo Simulations (D-F) in the hydrophobicslab. Coordinate sets of the toxins were kindly provided by Dr. Efremov [16]. The backbone of toxins is shown in ribbon model. Hydrocarbon core-penetrating residues are shown in stick model. Hydrocarbon core boundary at the extracellular side is indicated by red dots. The layer of lipid phosphates ("P") is shown by gold dots (at 5 Å outside the hydrocarbon boundary). The center of membrane is indicated by grey dots (at 15 Å inside the boundary). The orientations of each protein obtained by PPM and MC simulations are rather similar; and sets of membrane-penetrating residues are identical. However, the tilt between the protein axis and the membrane normal differ by 3° for 1rl3, by 10° for 1kxi and by 25° for 2cdx.

The first approach was applied here. It implements the implicit solvent approximation, which is based on the experimental linear relationship between the transfer energy and the accessible surface areas of solutes [79]. The required atomic solvation parameters have been derived from water-decadiene partition coefficients of organic molecules [27]. This method has a significant advantage: it operates directly with free energy of solvation, unlike molecular mechanics or electrostatic methods that include only the enthalpic component of free energy. Several versions of the implicit solvation model have been applied for positioning of α-helical peptides and transmembrane proteins in membranes [16]. However, this method has rarely been applied to peripheral proteins. Most notably, orientations of several snake venom cardiotoxins in the lipid bilayer have been simulated by Monte Carlo optimization with atomic solvation parameters that are different from ours. Coordinates of these cardotoxins were kindly provided by the authors [16], and thus can be compared with our results. This method is more computationally expensive because it refines the experimental 3D structures of the proteins, instead of keeping the initial structure, as in the present work. The simulated orientations of these toxins are similar to those in the present study (Figure 10). In particular, sets of membrane-penetrating residues are almost identical. A significant deviation in the tilt (~25°) was observed only for the cobra cardiotoxin CTXI (2cdx). This deviation may be caused by different conformations of the membrane-interacting loops in the original NMR model (used in this work), as compared with the conformation of the membrane-embedded neurotoxin obtained after its energetic refinement [16]; 2.7 Å r.m.s.d. of all C^α ^atoms).

The results of MD simulations with explicit water and lipids are also very similar to our results. For example, the orientations and membrane penetration depths of ovine prostaglandin H2 synthase 1 [12] and human secreted phospholipase A_2 _[13] obtained by MD simulations are close to those obtained by our method (OPM entries 1q4g and 1n28, Figure 6B). However, this comparison was based only on the published pictures of these proteins in membranes, because the MD-simulated coordinates of the proteins with lipids are unavailable.

The membrane-interaction regions of the proteins calculated by the electrostatic method were also rather similar to our results for three FYVE domains [21], the PH domain of phospholipase C [20], C2 domains [19], and phospholipase A_2 _[22]. This indicates that hydrophobic and electrostatic forces may actually work in concert to provide a stronger protein-membrane association. However, membrane penetration depths calculated by the electrostatic method were different by ~10–15 Å from the results obtained by all other methods, including MD [13], Monte Carlo simulations [16], and this work.

The electrostatic approach minimizes electrostatic energy of a protein at the charged planar membrane surface based on the finite difference Poisson-Boltzmann method [17-21]. This model omits hydrophobic interactions with the bilayer core and specific interactions with headgroups of lipids. Thus, it only includes a part of the *ΔG*_*head*-*group *_contribution in equation (1). It is assumed that the protein does not penetrate through the continuous van der Waals surface formed by headgroups of the lipids (although a few atoms can be artificially removed to allow some penetration into the interfacial rather than acyl chain region [22]). Therefore, in the electrostatic model, all proteins are located ~2 Å above the membrane surface. However, other computational methods demonstrate that these proteins pass through the interfacial region and penetrate the hydrophobic core by 1 to 6 Å, consistent with numerous experimental studies (EPR, fluorescence and others), positions of co-crystallized detergents, lipids, and Ca^2+ ^ions, and location of charged residues important for the protein binding inside the interfacial region (see Results).

## Conclusion

Our computational method for the positioning of proteins in membranes was successful for the set of 53 well-studied peripheral proteins. Therefore it was applied for the calculation of more than 470 membrane-associated proteins and peptides from the PDB. Here, for the first time, we have collected all peripheral proteins with known structures whose orientations have been experimentally studied *in vitro *and analyzed and classified a large and diverse set of peripheral proteins from the PDB. All these protein structures with calculated membrane boundaries are available through the OPM database.

Our calculations demonstrate the key role of non-specific hydrophobic and specific interactions with the lipids in the binding and arrangement of peripheral proteins in membranes. We find that most proteins examined not only interact with the membrane surface, but penetrate through the interfacial region and participate in the hydrophobic interactions with the hydrocarbon interior of membranes. This relatively deep penetration of peripheral proteins is consistent with experimental studies by spin-labeling, fluorescence, and NMR spectroscopy, significant contributions of exposed non-polar residues to membrane binding affinities as evaluated by mutagenesis, locations of crystallized lipids in the protein structures, and results of independent calculations with the hydrophobic slab model and MD simulations with explicit lipids.

## Methods

### Energy optimization

The computational approach for positioning of membrane proteins was previously descibed and implemented in program PPM 1.0. [26]. A protein was considered as a rigid body freely floating in the fluid hydrocarbon core of a lipid bilayer. Free energy of the protein (Δ*G*_calc_) represented a sum of transfer energies of all its atoms from water to the hydrocarbon core of the lipid bialyer (Δ*G*_*transf*_) and the ionization energies of charged residues (Δ*G*_*pK*_):

Δ *G*_*calc *_(*ϕ, τ, d*) = Δ *G*_*transf *_+ Δ *G*_*pK*_

The free energy difference was optimized in a coordinate system, in which Z axis is normal to the bilayer, and the origin is the bilayer center. This free energy difference depends on three variables (ϕ, τ, d); where *d *is the shift of the protein center along the Z axis relative to the xy plane, τ is the tilt angle of the longitudinal protein axis relative to the Z axis (membrane normal), and ϕ is the rotation angle that defines the direction of the tilt. The tilt (τ) of peripheral proteins was calculated as the angle between the bilayer normal and the molecular axis, relative to which the protein has the minimal moment of inertia [10]. This is different from the definition of longitudinal axis in transmembrane α-bundles and β-barrels as vector averages of transmembrane secondary structure vectors [26].

The energy of protein transfer from water to the lipid bilayer was calculated using the implicit solvation model:



where *ASA*_*i *_is the accessible surface area of atom *i*, and *σ*_*i*_^*W-M *^is the solvation parameter of atom *i *(its transfer energy from water to membrane interior expressed in kcal/mol per Å ^2^). *ASA *were determined using the subroutine SOLVA from NACCESS (provided by Dr. Hubbard) with van der Waals radii from Chothia [80], which implicitly take into account hydrogen atoms, and with the solvent probe radius of 1.4 Å.

All atomic solvation parameters were derived from the partition coefficients of organic compounds between water and decadiene [27]. These parameters were normalized by the effective concentration of water, which changes gradually in a narrow region between the lipid headgroup region and the hydrocarbon core. We used a sigmoid water concentration profile *f *(*z*_*i*_), as determined in EPR studies on spin-labeled phospholipids [25,81]:



The characteristic distance λ of this profile was chosen as 0.9 Å [25].

All charged residues of the protein were considered neutral in the membrane hydrocarbon core. The corresponding ionization energy was described by the Henderson-Hallelbalch equation, where the ionization energy of each residue *k *was distributed between its charged side-chain O or N atoms proportional to their relative accessible surface areas *ASA*_*i*_:



where *ASA*_*k*_^*tot *^is the total *ASA *of all charged atoms in the residue. pKa values of aspartate, glutamate, lysine, and aspargine residues were chosen as described previously [26]. The corresponding *ΔG*_*pK *_value for a histidine was zero, and the values for other residues were from 4 to 7 kcal/mol. Based on equation 5, the contributions of solvent-inaccessible charged groups were zero (such groups do not change their ionization state in the membrane). An ionizable group was treated as solvent-inaccessible if ASA of its polar atoms was less than 1 Å, or if it formed at least two hydrogen bonds in the protein structure.

Global energy minimization was performed by combining a grid scan and the Davidon-Fletcher-Powell method [26]. The lowest energy arrangement was always selected. Conformers of selected flexible side-chains located close to the water-membrane interface were adjusted as described previously [26]. Ligands were included only for membrane targeting domains co-crystallized with their lipid head group analogues (e.g. 1bwn, 1joc, or 1dsy). Then, acyl chains of bound lipids were reconstructed. A conformational search was conducted for five torsion angles in the lipid headgroup region (starting from P1-C2 bond) to identify conformers (± 60 or 180°) which do not produce interatomic hindrances and provide the lowest calculated transfer energies for each protein. The modeling was accomplished using QUANTA.

### Selection of well studied peripheral proteins

The set of peripheral proteins used for validation of our computational approach included all proteins with known 3D structures whose orientations in the lipid bilayer (53 proteins in Tables 1 and 2) or membrane binding affinities (38 proteins in Table 4) were experimentally evaluated *in vitro*, excluding studies conducted at non-physiologically low ionic strength. The selection was based on the following criteria: (1) 3D structures of the proteins represented the same domain or a combination of domains that were used in the binding studies, (2) potential membrane-interacting regions were present (not disordered or missing) in the crystal or NMR structures; (3) no significant conformational transitions or aggregation was experimentally detected during binding of the protein with lipid bilayers.

### Selection and analysis of membrane-associated proteins from the PDB

Identification and analysis of peripheral proteins from the PDB included the following six steps. First, all structures from the PDBSELECT90 set (~11,500 structures in release of November 2005, excluding viral particles) were optimized by our program PPM 1.0. A set of ~70 peptides was added, because PDBSELECT contains only polypeptide chains longer than 30 residues. Oligomeric structures were generated by Protein Quaternary Structure (PQS) server [82].

Secondly, all structures with calculated energies *(ΔG*_calc_) lower than -1 kcal/mol were selected and visually analyzed to eliminate proteins whose hydrophobic regions represented disordered loops with undefined spatial positions (usually in NMR models, at the N- or C-termini, or near regions with missing electron density).

Third, the remaining ~2700 structures were classified automatically to different SCOP families [59] based on the architecture of their largest membrane-associated domain.

Fourth, families whose proteins either had low transfer energies or potentially interacted with membranes (based on keyword search of PDB and SwissProt) were selected. The potential membrane-interacting regions were compared for all proteins in each family to define whether these regions represented the same or alternative binding modes in related proteins (alternative binding sites were detected in a relatively small number of cases, such as PX domains or small G-proteins).

Fifth, a quick analysis of UniProt and related PubMed records was conducted to define which structures could indeed associate with membranes, and retrieve their primary subcellular localization and topology. It was important to check if the obtained hydrophobic regions are known to be involved in association with other proteins *in vivo*, rather than interactions with lipid bilayers. For example, the "switch regions" of G-proteins, poly-Pro sequences in vinculin, and hydrophobic sites of extracellular domain of bone morphogenetic receptor are established protein-protein recognition motifs. All proteins with such regions were eliminated, except when these regions may be of "dual purpose", i.e. interact with bilayers as well as with other proteins *in vivo *(some toxins, polypeptide hormones, etc.). Finally, different PDB entries representing each selected protein were superimposed by the Secondary Structure Matching (SSM) server [83] to identify all significantly different structures, such as open and closed states of lipases. Also, the most functionally relevant quaternary structure of each complex was selected. This was usually the largest oligomeric complex (as defined by the PQS server), unless some data suggested otherwise. For instance, all phospholipases A_2 _and cytochromes P450 were taken as monomers, although some of them form dimers or trimers in crystals. Such oligomers may be stable in the crystal or even in aqueous solution, but they presumably dissociate in the membrane.

3D structures of identified peripheral proteins and membrane-associated peptides with calculated hydrophobic boundaries were deposited in the OPM database [1,24], with their calculated tilt angles, maximal membrane penetration depths, transfer energies, locations of hydrocarbon boundaries, spatial positions of all atoms in the membrane coordinate system, subcellular localization, topology, structural classification, and experimental verification data with PubMed references.

## Abbreviations

OPM, Orientations of Proteins in Membranes database; PPM, Positioning of Proteins in Membranes, (software); PDB, Protein Data Bank; SCOP, Structural Classification of Proteins database; SDSL, site-directed spin-labeling; PKC-C1, protein kinase C conserved 1; PKC-C2, protein kinase C conserved 2; PH, pleckstrin homology; EEA1-FYVE, Fab1, YOTB, Vac1 and Early endosomal antigen 1, PX, phox; ENTH, Epsin N-terminal Homology; ANTH, AP180 N-terminal Homology; FERM, Band 4.1, Ezrin, Radixun, Moesin; cPLA_2_; cytosolic phospholipase A_2_; sPLA_2_, secreted phospholipase A_2_; sytI, synaptagmin I; PI, phosphatydylinositol, PS, phosphatidylserine, PC, phoshatidylcholine; PE, phosphatidylethanolamine; PMA, phorbol 12-myristate 13-acetate; PI(4,5)P_2_, phosphatidylinositol-4,5-diphosphate; PI(3,4)P_2_, phosphatidylinositol-3,4-diphosphate; PI(3)P, phosphatidylinositol-3-monophosphate; PI(4)P, phosphatidylinositol-4-monophosphate.

## Authors' contributions

ALL developed the method and software, calculated the positions of proteins in membranes, analyzed the results, and maintained OPM database; IDP participated in analysis and interpretation of results, compared them with published experimental studies, and created figures, MAL developed and maintained the OPM database. All authors participated in the manuscript preparation and approved the final manuscript. Authors declare that they have no competing interest.

## Supplementary Material

Additional file 1 **A Table**. Calculated membrane penetration depths (*D*, Å) and binding energies (Δ*G*_*calc*_, kcal/mol) of selected monotopic and peripheral proteins included in the OPM database. Click here for file
