# Supplementary material for: The role of hydrophobic interactions in positioning of peripheral proteins in membranes
Source: BMC Struct Biol. 2007 Jun 29;7:44. doi: 10.1186/1472-6807-7-44 (PMC1934363; doi:10.1186/1472-6807-7-44)
Supplement: Additional file 1 — A Table. Calculated membrane penetration depths (D, Å) and binding energies (ΔGcalc, kcal/mol) of selected monotopic and peripheral proteins included in the OPM database. [file 1472-6807-7-44-S1.doc]

**Table 1.** Calculated membrane penetration depths (*D*, Å) and transfer energies (Δ*G_calc_*, kcal/mol) of selected monotopic and peripheral proteins included in the OPM database.

| **Proteins and PDB ids of unique structures^a^** | **Δ*G_calc_*** | **D** | **Membrane anchoring structures ^b^** |
| --- | --- | --- | --- |
| **A. Peripheral domains of integral transmembrane proteins** | | | |
| Monoamine oxidases A and B (1o5w, 1ojd, 2bxs) | -9.3 to -30.5 | 9.6 to 33.0 | α+L+1TMH |
| Fatty acid amine hydrolase (1mt5) | -30.8 | 10.0 | α+(1TMH) |
| Cytochromes P450 (1dt6, 1nr6, 1og5, [1po5](http://opm.phar.umich.edu/protein.php?pdbid=1po5), 1pq2, 1r9o, 1tqn, 1suo, 1w0f, 1z10, 2bdm, 2f9q) | -6.9 to -20.7 | 4.7 to 12.5 | α+L+(2TMH) |
| Corticosteroid 11β-dehydrogenases (1y5m, 1xu7) | -14.9, -13.4 | 3.6, 7.8 | L+(1TMH) |
| Signal peptidase (1t7d, 1kn9, 1b12) | -2.4 to -5.9 | 2.4 to 4.5 | β-sheet +(2TMH) |
| Membrane protease specific for a stomatin homolog (2deo) | -5.4 | 4.2 | α+(4TMH) |
| Signal recognition particle receptor (1nrj) | -4.9 | 3.5 | L+(1TMH) |
| Emerin (1jei) | -3.8 | 6.3 | L+(1TMH) |
| Mitochondria fission protein Fis1 (1nzn) | -5.6 | 6.1 | α+(1 TMH) |
| T-cell surface glycoprotein CD1d antigen (1z5l, 1zhn) | -3.0, -3.6 | 2.4, 2.3 | L+(1 TMH) |
| [Immunodominant region of protein G of BRSV](http://opm.phar.umich.edu/families.php?superfamily=158) (1brv) | -4.5 | 7.5 | α+L+(1 TMH) |
| N-terminal domain of follicle-stimulating receptor (1xwd) | -5.0 | 3.1 | L+(7 TMH +Ac) |
| N-terminal domain of cholecystokinin A receptor (1d6g) | -16.1 | 8.0 | L+(7 TMH +Ac) |
| Major envelope glycoprotein E (1ok8) | -9.9 | 4.9 | L+(5 TMH) |
| **B. Integral monotopic proteins** | | | |
| Prostaglandin H2 synthases 1 and 2 (**1q4g** and1cx2) | -37.8, -38.3 | 7.2, 10.4 | α |
| Lanosterol synthase (1w6k) and squalene-hopene cyclase (2sqc) | -19.9, -23.8 | 6.5, 7.2 | α |
| Microsomal prostaglandin E synthase (1z9h) | -13.1 | 4.4 | α |
| Carnitine O-palmitoyltransferase 2 (2h4t) | -8.6 | 3.6 | α |
| **C. Peripheral proteins** | | | |
| **C.1. Enzymes** | | | |
| Bile-salt activated lipases (1akn, 1aql, 1f6w) | -4.4 to -9.7 | 5.4 to 6.0 | L |
| Gastric lipase (1hlg, 1k8q) | -5.0, -10.8 | 3.4, 6.3 | α+L |
| Fungal lipases (1dt5, 1gz7, 1ein, 1lpp, 1lbs, 1lgy, 1thg, 1tib, 1trh, 3tgl, 4tgl) | -3.3 to -30.6 | 1.9 to 11.5 | α+L |
| Bacterial lipases (1cvl, 1ex9, 1isp, 1jfr, 1ji3, 1ku0, 1qge, 5lip) | -2.6 to -38.2 | 1.8 to 11.4 | α+L |
| Type-B carboxylesterase/lipase (1cle) | -28.0 | 9.6 | α+L |
| Pancreatic lipases (1bu8, **1eth,** 1gpl, 1hpl, 1lpa, 1lpb, 1n8s) | -5.2 to -26.2 | 3.3 to 13.4 | α+L |
| Palmitoyl protein thioesterases (1eh5, 1pja) | -5.1, -2.8 | 4.6, 3.9 | α+L |
| Cutinase (1oxm) | -10.4 | 7.4 | L |
| Cholesterol oxidases (1b4v, **1coy**) | -7.1, -4.1 | 5.7, 5.3 | α+L |
| Carotenoid oxygenase (2biw) | -12.7 | 5.1 | α+L |
| Phospholipases A_2_ (1ae7, 1bk9, 1bjj, 1g2x, 1g4i, 1gmz, 1god, 1gp7, 1jia, 1jlt, 1kp4, **1le6,** 1m8t, 1mc2, **1n28**, 1oz6, 1ozy,1pp2, **1poa**, **1poc**, 1s6b, 1s8i, 1tc8, **1vap,** 1vip, 1u4j, 1umv, 1xxs, 1zwp, 4p2p, 5p2p) | -1.8 to -21.8 | 1.4 to 7.7 | α+L |
| Lipoxygenases ^d^ (**1lox, 1zq4)** | -7.4, -5.0 | 6.3, 5.9 | α+L |
| α-Toxins ^d^ (**1olp, 1gyg**, 1kho, **1ca1**) | -1.4 to -4.8 | 1.3 to 5.5 | L |
| Phospholipases C ^d^ (1aod, 1djx, 1gym, 2plc, **2ptd**) | -3.2 to -8.0 | 3.9 to 7.4 | α+L |
| Cytosolic phospholipase A_2_ ^d^ (1cjy) and patatin (1oxw) | -9.8, -6.5 | 5.7, 4.3 | α+L |
| Sphingomyelinase C (1zwx) | -6.2 | 6.0 | L |
| Transglycosidases (1ogs, 1vff) | -4.1, -6.5 | 3.2, 3.3 | L |
| Ferro[chelatase](http://opm.phar.umich.edu/families.php?superfamily=137) (1hrk) | -9.2 | 7.2 | α+L |
| Myotubularin-related protein 2 (1zvr) | -2.3 | 2.9 | α+L |
| Cholinesterases (1ea5, 1n5m, 1f8u, 1p0i) | -2.6 to -6.4 | 1.5 to 3.4 | α+L+(GPI) |
| Glycosyltransferase MurG (1f0k, 1nlm) | -6.9, -7.5 | 4.2, 4.5 | α+L |
| Micobacterial antigens (1sfr, 1f0n, 1dqz) | -5.3 to -6.0 | 2.6 to 4.5 | α+L |
| Dihydroorotate dehydrogenases (1d3h, 1f76, 1uum) and glycolate oxidase (1gox) | -4.5 to -9.5 | 3.0 to 10.2 | α+L |
| [Vitelline membrane outer protein-I](http://opm.phar.umich.edu/families.php?superfamily=113) (1vmo) | -3.4 | 3.0 | L |
| [Colicin E3](http://opm.phar.umich.edu/families.php?superfamily=53) (1jch) | -9.0 | 2.7 | L |
| **C.2. Water-soluble carriers of nonpolar substances** | | | |
| [Glycolipid transfer protein](http://opm.phar.umich.edu/families.php?superfamily=92)s (1tfj, 1swx, 1sx6) | -4.9 to -7.6 | 2.8 to 3.9 | α+L |
| [Lipocalins](http://opm.phar.umich.edu/families.php?superfamily=52) (1aqb, 1b56, 1bwy, 1cbr, 1cbs, 1crb, 1fdq, 1ftp, 1g7n, 1ggl, 1hmt, 1icm, 1iiu, 1kqw, 1kt6, 1kzw, 1lpj, 1o8v, 1pmp, 1qwd, 1rbp, 1rlb, 1tow, 1vyf) | -2.2 to -5.9 | 1.6 to 6.9 | α+L |
| Polyisoprenoid-binding protein (1wub) | -5.6 | 3.7 | L |
| [GM2 activator](http://opm.phar.umich.edu/families.php?superfamily=114)s (1pub, 1tjj, 2agc, 2ag4) | -4.7 to -9.3 | 4.2 to 5.2 | β-hairpin +L |
| α-Tocopherol (1oiz, 1r5l) and phosphatidylinositol sec14p (1aua) transfer proteins | -10.7 to -20.7 | 4.0 to 8.4 | α |
| [Sterol carrier protein](http://opm.phar.umich.edu/families.php?superfamily=144) (1c44) | -3.2 | 4.0 | α+L |
| Phosphatidylinositol transfer proteins (1t27, 1uw5) and STAR domains (1jss, 1ln1, 1em2) | -2.2 to -4.9 | 2.6 to 4.7 | α+L |
| Oxysterol-binding protein (1zi7) | -5.9 | 3.0 | L |
| **C.3. Membrane-targeting and other structural domains** | | | |
| [C2 domain](http://opm.phar.umich.edu/families.php?superfamily=47)s (1a25, 1bdy, 1byn, 1d5r, 1dqv, **1dsy,** 1gmi, **1rlw**, **1rsy,** 1ugk, **1uov,** 2b3r, 2bwq) | -1.3 to -7.1 | 1.2 to 5.3 | L+lip |
| C2 domains of blood coagulation factors (1czs, **1d7p**,**1sdd**) | -3.0 to -5.6 | 3.3 to 4.2 | L+ lip |
| [PX domain](http://opm.phar.umich.edu/families.php?superfamily=60)s (**1h6h,** 1kmd, **1kq6, 1o7k**, 1ocu, 1ocs) | -1.9 to -6.5 | 1.4 to 3.3 | L+ lip |
| [C1 domain](http://opm.phar.umich.edu/families.php?superfamily=63)s (**1faq**, 1kbf, **1ptr**, 1r79, **1tbn**) | -2.3 to -8.1 | 2.4 to 7.5 | L+ lip |
| FYVE domains (**1hyi, 1vfy**, 1joc) | -2.9 to -4.0 | 2.5 to 2.9 | L+ lip |
| PH domains (1bwn, 1dbh, 1dyn, 1eaz, 1fao, 1foe, 1mai, 1nty, 1p6s, 1pls, 1w1g, 1unq, 1v5u, 2bcj) and disabled homolog 1 (1nu2) | -2.0 to -11.2 | 1.6 to 5.0 | L+ lip |
| [ENTH (**1h0a),** VHS (1dvp), and CALM (1hfa) domain](http://opm.phar.umich.edu/families.php?superfamily=39)s | -3.2 to -6.5 | 2.6 to 4.0 | α+L+lip |
| Tubby protein (1i7e, 1c8z) | -3.6, -4.7 | 2.6, 6.2 | L+lip |
| [Annexins](http://opm.phar.umich.edu/families.php?superfamily=43) (1a8a, 1ann, 1axn, 1dk5, **1dm5**, 1hm6, **1hvf**, 1ia4, 1m9i, 1n00, 1w3w, 1w7b, 1yii) | -1.2 to -8.3 | 1.7 to 3.4 | L+ lip |
| [GLA-domain](http://opm.phar.umich.edu/families.php?superfamily=97)s (**1dan**, **1lqv,** 1nl2, **1pfx)** | -3.8 to -6.7 | 3.2 to 4.8 | α + lip |
| [Influenza virus matrix protein M1](http://opm.phar.umich.edu/families.php?superfamily=44) (1aa7) | -3.7 | 2.5 | L |
| Hisactophilin-1 (1hce) | -1.2 | 1.9 | L + (Ac) |
| Seminal plasma protein (**1h8p**) | -12.3 | 9.2 | L + lip |
| Translocation ATPase SecA (1tf5) | -2.6 | 2.4 | α+L |
| Exocyst complex component Sec5 (1uad) | -2.2 | 2.0 | L+(Ac) |
| Synapsin I (**1auv)** | -4.4 | 2.5 | α+L |
| Epididymal secretory protein E1 (1nep) and Rho GDP-dissociation inhibitors (1ds6, 1hh4, 1qvy) | -3.5 to -5.3 | 2.8 to 7.7 | β-hairpin +L+(Ac) |
| Rab GDP dissociation inhibitor alpha (1d5t) | -2.4 | 2.4 | L |
| Phosducin (1a0r, 2trc, 1b9x) | -1.4 to -3.7 | 1.6 to 4.4 | α+L+Ac |
| Spectrin (2spc) and α-actinin-2 (1hci) | -3.5, -3.4 | 1.6, 1.3 | α |
| Peroxin pex5 (1hxi) and vesicular transport protein sec17 (1qqe) | -4.9, -7.1 | 6.8, 7.9 | α |
| **C.4. Electron carriers** | | | |
| [Cytochromes c](http://opm.phar.umich.edu/families.php?superfamily=78) (1a8c, 1c6s, 1co6, 1cor, 1f1f, **1hrc**, 1kx7, 1ls9, 1m70, 451c) | -1.2 to -7.5 | 1.1 to 4.8 | L |
| [Cupredoxins](http://opm.phar.umich.edu/families.php?superfamily=101) (1b3i, 1bxv, 1cuo, 1f56, 1id2, 1pmy, 1rkr, 1sfd, 2plt, 9pcy) | -1.3 to -5.3 | 1.1 to 3.6 | L |
| [High potential iron protein](http://opm.phar.umich.edu/families.php?superfamily=124) (1hpi) | -4.7 | 3.8 | α+L |
| Adrenodoxin reductase (1e6e) | -4.3 | 4.2 | α |
| Electron transfer flavoproteins (1efp, 1efv) | -4.7, -3.1 | 3.8, 2.9 | α |
| **C.5 Polypeptide ligands (hormones, inhibitors, toxins, and antimicrobial peptides)** | | | |
| [α-Helical peptide hormones](http://opm.phar.umich.edu/families.php?superfamily=154) (1gcn, **1icy**, 1lbj, 1p9f, 1smz, 1wso) | -3.4 to -11.2 | 4.2 to 9.6 | α |
| [Tachykinin peptides](http://opm.phar.umich.edu/families.php?superfamily=152) (1mxq, 1myu, 1n6t) | -4.0 to -6.3 | 3.6 to 9.9 | α |
| [Octreotide](http://opm.phar.umich.edu/families.php?superfamily=167) (**1soc**) | -5.6 | 3.1 | β-hairpin |
| Saposin B (1n69), and NK-lysin (1nkl) | -3.1, -4.8 | 1.6, 3.7 | L +lip |
| [Heat-stable enterotoxin B](http://opm.phar.umich.edu/families.php?superfamily=96) (1ehs) | -4.5 | 2.8 | α+L |
| Conotoxins (1fu3, 1fyg, 1g1z, 1rmk, 1f3k), spider toxins (1agg, **1d1h,** 1qdp, 1kqi, 1vtx, 1qk6, 1s6x, 1v7f), insect toxins (1lmr), albumin 1 (1p8b), and leginsulin (1ju8) | -2.5 to -16.9 | 2.6 to 18.3 | β-hairpin+L |
| Scorpion toxins (1cn2, 1djt, 1dq7, 1fh3, 1jza, 1kv0, 1npi, 1sis, **2crd,** 2sn3) | -1.6 to -7.4 | 1.2 to 7.3 | L |
| Snake venom toxins (1cdt, **1ffj**, **1h0j**, 1hc9, 1kxi, **1tgx**,1txa, 1ug4, 1drs) | -3.2 to -18.1 | 3.0 to 8.7 | β-sheet+L |
| [Neurotoxin III](http://opm.phar.umich.edu/families.php?superfamily=122) (1ans) | -3.3 | 2.4 | L |
| [Defensin](http://opm.phar.umich.edu/families.php?superfamily=56)s (1bnb, 1dfn, 1e4r, 1e4t, 1ews, 1ijv, 1kj6, 1ut3) and sea anemone sodium channel toxins (1ahl, 1apf, 1atx, 1bds, 1shi) | -2.9 to -16.0 | 2.5 to 11.5 | β-sheet+L |
| Poneratoxin (1g92) | -23.0 | 17.9 | α+L |
| α-[Conotoxins](http://opm.phar.umich.edu/families.php?superfamily=157) (1akg, 1dg2, 1mii, 1pen) | -4.0 to -6.9 | 2.6 to 5.2 | α |
| Subtilosin (**1pxq)** and microcin J25 (1q71, 1s7p) | -6.3 to -7.4 | 5.1 to 16.3 | β-hairpin + α |
| [Tricyclic peptide RP71935](http://opm.phar.umich.edu/families.php?superfamily=166) (1rpb) | -9.5 | 4.6 | L |
| [Gramicidin S](http://opm.phar.umich.edu/families.php?superfamily=169) (**1tk2)** | -14.1 | 13.0 | β-haipin |
| Antimicrobial peptide HP (1p0l) | -6.3 | 4.0 | α |
| Lactoferricin B (**1lfc**) | -4.6 | 5.3 | β-hairpin |
| Daptomycin (1t5m, **1t5n**, 1xt7) and tsushimycin (1w3m) | -7.2 to -14.8 | 3.3 to 5.4 | L+Ac |
| [Cyclotides](http://opm.phar.umich.edu/families.php?superfamily=64) (1bh4, 1df6, 1kal, **1nb1,** 1orx, 1pt4, 1r1f, 1vb8) | -3.8 to 12.7 | 3.4 to 9.8 | β-sheet+L |
| [Leucocin-like bacteriocin](http://opm.phar.umich.edu/families.php?superfamily=150)s (1cw6, 1ohm) | -5.6, -2.2 | 4.0, 2.3 | α+L |
| **C.6. Channel-forming polypeptides** | | | |
| Apoptosis regulator Bcl-2 (1g5m) | -3.9 | 4.6 | α |
| [Colicin](http://opm.phar.umich.edu/families.php?superfamily=93) A (1col) | -1.5 | 1.0 | L |
| [δ-Endotoxin](http://opm.phar.umich.edu/families.php?superfamily=95)s (1dlc, 1i5p, 1ji6, 1w99) | -1.3 to -7.2 | 1.6 to 11.7 | α+L |
| [Anemone pore-forming cytolysin](http://opm.phar.umich.edu/families.php?superfamily=103)s (**1iaz**,1gwy) | -2.2,-3.6 | 3.1, 4.3 | L |
| [Perfringolysin](http://opm.phar.umich.edu/families.php?superfamily=116) (**1pfo**) | -5.5 | 3.4 | L |
| Botulinum neurotoxin B (1epw) | -4.5 | 3.3 | L |
| Crambin (1ejg), β-purothionin (1bhp), and hellethionin (1nbl) | -3.5 to -7.0 | 2.7 to 3.2 | α+L |
| [Bacteriocin AS-48](http://opm.phar.umich.edu/families.php?superfamily=84) (1o82) | -6.8 | 8.1 | α |
| Ectatomin (1eci) | -2.9 | 4.1 | α+L |
| [Magainin](http://opm.phar.umich.edu/families.php?superfamily=68) (**2mag**) | -14.5 | 10.1 | α |
| [Peptaibol](http://opm.phar.umich.edu/families.php?superfamily=76)s (**1amt,** 1ee7, **1ih9**, **1joh,** 1m24, 1ob4, 1ob6, 1ob7) | -14.3 to -20.9 | 9.9 to 28.1 | α |
| Insect defensins (1l4v,1ozz, 1myn) | -6.6 to -6.8 | 4.5 to 6.1 | α+L |
| Plant defensins (1ayj, 1jkz) | -8.9,-4.1 | 11.3, 3.3 | L |
| Moricins (1kv4, 1x22) | -11.9, -10.9 | 7.2, 4.2 | α |
| Pleruocidin (1z64) | -13.8 | 10.7 | α |
| Actagardine (1aj1), mersacidin (1mqx, 1mqy, 1mqz, 1qow), and nisin (**1wco)** | -2.3 to -9.5 | 2.4 to 11.5 | L+**Lip** |

^a^ Each PDB file represents a different protein or a different conformational state of the same protein. Related proteins are combined together. PDB codes are shown in bold for proteins whose membrane binding was studied quantitatively *in vitro* (Tables 1-3). PDB codes are underlined if the calculated membrane boundaries could be verified based on locations of ligands, detergents, or lipidated residues in the experimental structure.

^b^ “α” – surface α-helix, “β” - β-structure, “L” - loops, “lip” – non-covalently bound lipid that serves as an anchor rather than as a substrate or a transported molecule, “Ac”– covalently bound hydrophobic moiety, TMH – transmembrane α-helix. Membrane-anchoring elements are indicated in parentheses were not present in the structures but indicated in UniProt or found in the literature. Information about transmembrane α-helices in the parentheses was taken from UniProt. Some of these α-helices are tentative or theoretically predicted.

^d^ Classification of phospholipases was based on structures of their catalytic membrane-bound domains. Pancreatic lipases, lipoxigenases and α-toxins have a common regulatory PLAT domain that also associates with membranes and may relate to C2 domains.
